# Bone Morphogenetic Protein Pathway Antagonism by *Grem1* Regulates Epithelial Cell Fate in Intestinal Regeneration

**DOI:** 10.1053/j.gastro.2021.03.052

**Published:** 2021-04-02

**Authors:** Martijn A. J. Koppens, Hayley Davis, Gabriel N. Valbuena, Eoghan J. Mulholland, Nadia Nasreddin, Mathilde Colombe, Agne Antanaviciute, Sujata Biswas, Matthias Friedrich, Lennard Lee, Oxford IBD Cohort Investigators, Lai Mun Wang, Viktor H. Koelzer, James E. East, Alison Simmons, Douglas J. Winton, Simon J. Leedham

**Affiliations:** 1 Intestinal Stem Cell Biology Lab, Wellcome Centre Human Genetics, University of Oxford, Oxford, United Kingdom; 2 Li Ka Shing Centre, Cancer Research UK Cambridge Institute, University of Cambridge, Cambridge, United Kingdom; 3 Medical Research Council Human Immunology Unit, Medical Research Council Weatherall Institute of Molecular Medicine, John Radcliffe Hospital, University of Oxford, Oxford, United Kingdom; 4 Medical Research Council Weatherall Institute of Molecular Medicine Centre for Computational Biology, Medical Research Council Weatherall Institute of Molecular Medicine, John Radcliffe Hospital, University of Oxford, Oxford, United Kingdom; 5 The Kennedy Institute of Rheumatology, Nuffield Department of Orthopaedics, Rheumatology and Musculoskeletal Science, University of Oxford, Oxford, United Kingdom; 6 Cancer Genetics and Evolution Laboratory, Institute of Cancer and Genomic Sciences, College of Medical and Dental Sciences, University of Birmingham, Birmingham, United Kingdom; 7 Department of Laboratory Medicine, Changi General Hospital, SingHealth, Singapore, Singapore; 8 Department of Pathology and Molecular Pathology, University Hospital Zürich, Zürich, Switzerland; 9 Department of Oncology and Nuffield Department of Medicine, University of Oxford, Oxford, United Kingdom; 10 Translational Gastroenterology Unit, John Radcliffe Hospital, University of Oxford, and Oxford National Institute for Health Research Biomedical Research Centre, Oxford, United Kingdom

**Keywords:** Intestinal Regeneration, Dedifferentiation, Bone Morphogenetic Protein, Grem1

## Abstract

**Background & Aims:**

In homeostasis, intestinal cell fate is controlled by balanced gradients of morphogen signaling. The bone morphogenetic protein (BMP) pathway has a physiological, prodifferentiation role, predominantly inferred through previous experimental pathway inactivation. Intestinal regeneration is underpinned by dedifferentiation and cell plasticity, but the signaling pathways that regulate this adaptive reprogramming are not well understood. We assessed the BMP signaling landscape and investigated the impact and therapeutic potential of pathway manipulation in homeostasis and regeneration.

**Figure:**
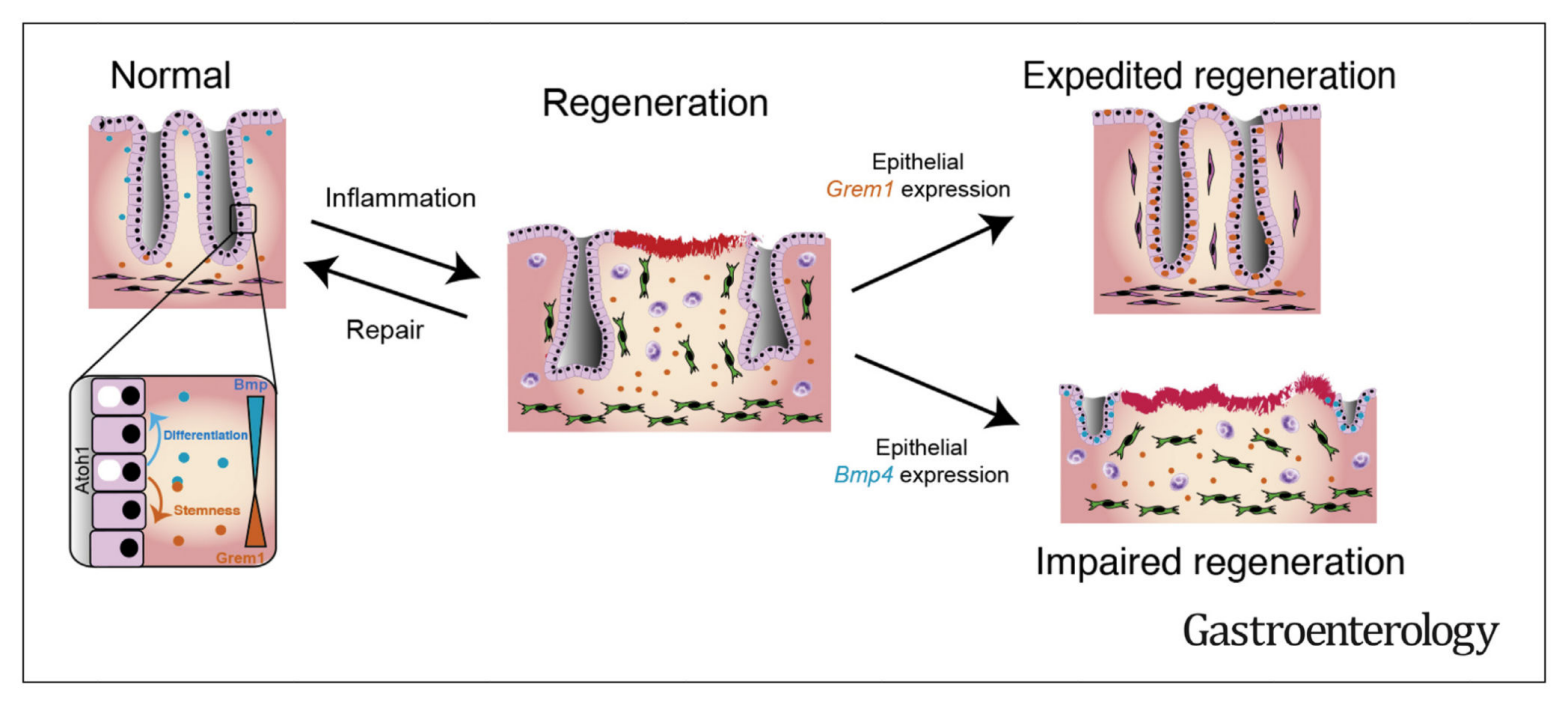

**Methods:**

A novel mouse model was generated to assess the effect of the autocrine Bmp4 ligand on individual secretory cell fate. We spatio-temporally mapped BMP signaling in mouse and human regenerating intestine. Transgenic models were used to explore the functional impact of pathway manipulation on stem cell fate and intestinal regeneration.

**Results:**

In homeostasis, ligand exposure reduced proliferation, expedited terminal differentiation, abrogated secretory cell survival, and prevented dedifferentiation. After ulceration, physiological attenuation of BMP signaling arose through upregulation of the secreted antagonist *Grem1* from topographically distinct populations of fibroblasts. Concomitant expression supported functional compensation after *Grem1* deletion from tissue-resident cells. BMP pathway manipulation showed that antagonist-mediated BMP attenuation was obligatory but functionally submaximal, because regeneration was impaired or enhanced by epithelial overexpression of *Bmp4* or *Grem1,* respectively. Mechanistically, *Bmp4* abrogated regenerative stem cell reprogramming despite a convergent impact of YAP/TAZ on cell fate in remodeled wounds.

**Conclusions:**

BMP signaling prevents epithelial dedifferentiation, and pathway attenuation through stromal Grem1 upregulation was required for adaptive reprogramming in intestinal regeneration. This intercompartmental antagonism was functionally submaximal, raising the possibility of therapeutic pathway manipulation in inflammatory bowel disease.

The intestinal mucosa is an ideal tissue for the study of homeostatic, regenerative, and pathologic epithelial cell fate determination because it has stereotypical crypt-based architecture, well-defined stem cell markers, and clear human disease implications. Secreted cell-signaling networks generate mucosal gradients that regulate the phenotypic response of epithelial cells migrating along the crypt.^1,2^ Bone morphogenetic protein (BMP) signaling is a key pathway, with a polarized gradient, maximal at the luminal surface, established through inter-compartmental cross talk enabled by differential expression of ligands, receptors, and antagonists.^3,4^ BMP ligands are predominantly secreted by the intercrypt mesenchyme and act on epithelial cells to promote differentiation. At the crypt base, exclusive expression of paracrine ligand-sequestering BMP antagonists (eg, gremlin 1, gremlin 2, chordin-like 2) from the muscularis mucosae restricts BMP activity and promotes epithelial stem cell activity within the crypt basal niche.

Although the physiologic expression patterns of BMP pathway constituents have been mapped in vivo,^3,4^ the homeostatic prodifferentiation function of BMP has been predominantly inferred through pathway inactivation. Epithelial BMP receptor knockout^5^ or antagonist knock in^6,7^ disrupts epithelial cell fate determination and induces tumorigenesis through promotion of an aberrant stem/progenitor phenotype. The importance of BMP antagonism in promoting stem cell function was exploited in organoid system development, which requires supraphysiological media concentrations of BMP antagonists for successful culture.^8^ This introduces challenges for the use of organoids in assessing the nuanced physiological role of the BMP ligands.

The complexity of morphogen signaling networks permits amplification or attenuation of the biological effects of individual pathways in a dynamic and context-dependent manner. Disruption in the homeostatic morphogen balance initiates the profound multi-compartmental response to injury and underpins the regenerative capacity of the intestinal epithelium. After injury, epithelial denudation skews homeostatic epithelial-mesenchymal cross talk and induces a localized immune response.^9^ Mucosal inflammation provokes dysregulation of the fibroblastic niche, with activation of diverse stromal cell populations.^10^ At a cellular level, this temporary signaling instability induces epithelial cell dedifferentiation,^11^ activation of regenerative stem cells,^12,13^ and promotion of fetal epithelial cell reprogramming, partly mediated by YAP/TAZ signaling.^14,15^ The mechanisms that coordinate this profound adaptive cell reprogramming response are not well understood, and the importance of the BMP pathway is not established.

Here, we assess the impact of BMP ligand manipulation on homeostatic epithelial cell fate, spatiotemporally map the mucosal BMP signaling landscape in intestinal regeneration, and assess the functional importance of BMP signaling in regulating effective wound healing.

## Materials and Methods

### Animal Models

All animal experiments were performed in accordance with the guidelines of the United Kingdom Home Office, under the authority of a Home Office project license approved by the Animal Welfare and Ethical Review Body at the Wellcome Centre for Human Genetics, University of Oxford. All mice were housed in a specific-pathogen-free facility, with unrestricted access to food and water. All strains used in this study were maintained on the C57BL/6J background for ≥6 generations. All procedures were performed in male and female mice of at least 6 weeks of age.

### *Generation of* Rosa26^Bmp4^ *Mice*

To generate *Rosa26*^*Bmp4*^ mice, a Bmp4 complementary DNA cassette was cloned into the integrase mediated cassette exchange vector (CB93) and transfected into RS-PhiC31 ES cells. Recombinant clones were obtained which harbor the Bmp4 complementary DNA transgene positioned within the Rosa26 locus, allowing for Cre-dependent activation of transgene expression. Recombinant clones were injected into blastocysts, and chimeras were generated (Wellcome Centre for Human Genetics Trangenics Core). Chimeras were crossed with wild-type C57BL/6J mice to obtain F1 heterozygotes. The following primers were used for genotyping (forward rbGpaF1 CAGCCCCCTGCTGTCCATTCCTTA, reverse Rosa3HR CGGGA-GAAATGGATATGAAGTACTGGGC) and (forward Caggs CAGCCATTGCTTTTATGGT, reverse Ex Neo2 GTTGTGCCCAGTCATAGCCGAATAG).

### Treatment of Animals

All experimental mice were from a C57/BL6 background, backcrossed for at least 6 generations, and were housed in specific-pathogen-free cages. Induction of CreER^T2^ in animals was performed out using the free base tamoxifen (Sigma-Aldrich, St. Louis, MO) dissolved in ethanol/oil (1:9). To obtain *Grem1* knockout mice, 6-week-old *Cagg-CreER*^*T2*^;*Grem1^fl/fl^* mice were injected with tamoxifen intraperitoneally, 1 mg daily, for 5 days. For lineage tracing after biopsy wounding (*Atoh1CreER^T2^*;*Rosa26^tdTomato^*), recombination was induced by a single dose of 3 mg of tamoxifen, and biopsy wounding was performed 24 hours after the tamoxifen injection. The biopsy-wounding procedure was performed on isoflurane-anesthetized mice, using a miniature rigid endoscope (1.9 mm outer diameter; Karl Storz, Berkshire, United Kingdom), inserted 2 to 3 cm into the rectum. Three biopsy samples were taken from well-separated areas in the colon wall using forceps (1 mm, 3F). The mice were killed at various times after wounding.

Small intestinal injury was induced by exposing animals to whole-body irradiation (10 Gy) performed using an IBL-637 irradiator with a caesium-137 source. Irradiation was delivered over 652 seconds at 0.92 Gy/min.

For dextran sodium sulfate (DSS)-induced colitis, litter-mates were selected for treatment, where possible, to minimize the impact of microbiome differences on colitis progression. Mice were treated with drinking water containing 1.5% DSS (MP Biomedicals, Irvine, CA) for 5 days, after which their water was changed back to normal water to recover for 1 day, after which they were humanely killed. The mice were weighed daily and checked for signs of discomfort (eg, hunching, pale feet). The mice were killed when their weight dropped <80% of their weight at the start of the DSS treatment or when they showed other clear symptoms of discomfort.

### Human Samples

Human pathologic samples used were from normal tissue distant to tumor resections or from ulcerative colitis specimens taken from bowel resections for acute severe colitis after ethical approval and individual informed consent (MREC 16/YH/0247).

### RNA Extraction, Gene Expression Analysis, and RNA Sequencing

RNA extraction and quantitative reverse-transcription polymerase chain reaction gene expression was undertaken using standard techniques described in detail in the supplementary-material Methods. RNA sequencing (RNAseq), using standard techniques described in detail in Supplementary Methods, was undertaken on colon ulcers and distant normal colon tissue excised from biopsy-wounded wild-type and *Vil1-Cre*;*Rosa26^Bmp4^* mice at 1 day and 3 days after wounding.

### Bioinformatic Analysis of RNA Sequencing

RNAseq data from inflamed colon tissue from patients with inflammatory bowel disease (IBD) and noninflamed colon tissue from controls without IBD16 were downloaded from the European Nucleotide Archive (accession number PRJNA326727). Raw sequence reads were subjected to adapter trimming using *BBduk* (BBTools 38.46; https://sourceforge.net/projects/bbmap/files/). Trimmed reads were aligned to the Genome Reference Consortium Human genome build 38 (GRCh38) of the human reference (for human colon tissue data) or to the GRCm38 build of the mouse reference (for mouse ulcer data) using STAR 2.7.0f (https://github.com/alexdobin/STAR). Ensembl 96 (https://www.ensembl.org/index.html?redirect=no) annotations were used for alignment and subsequent quantifications. Gene expression was quantified using RSEM 1.3.1 (https://github.com/deweylab/RSEM/releases/tag/v1.3.1). The processed RNAseq data were analyzed in R 3.6.1 software (R Foundation for Statistical Computing, Vienna, Austria). Differential expression analyses were performed using the *limma* 3.40.6 package. Gene Set Enrichment Analyses were performed using the *fgsea* 1.10.1 package. The YAP/TAZ gene signature includes genes that had >2-fold upregulation between wild-type and YAP-overexpressing intestinal epithelial cells,14 whereas the fetal intestinal gene signature includes genes that were significantly upregulated >2-fold (false discovery rate <0.05) in fetal intestinal epithelial cell cultures over adult intestinal epithelial cell cultures.^15^

### Tissue Preparation and Staining

Either the entire intestinal tract was removed and divided into small intestine (proximal, middle, and distal) and colon, or in case of wounding/colitis experiments, only the colon was removed. The intestines were flushed with phosphate-buffered saline, opened longitudinally using a gut preparation apparatus, and fixed overnight in 10% neutral buffered formalin (Merck, Readington, NJ). The tissues were then embedded in paraffin and sectioned at 4 *μ*m using a microtome (Leica, Buffalo Grove, IL) with diethylpyrocarbonate-treated water (Merck) in the water bath.

To find the ulcers generated by biopsy wounding, the entire tissue block was sectioned, and 4 serial sections collected, followed by a 50-*μ*m trim (discarded), throughout the block. H&E staining was performed on 1 of 4 sections of each series following standard procedures, leaving 3 blank slides per series for other stains. The mid-ulcer series of sections was determined for all ulcers by identifying their range within the entire set of H&E slides per block. For whole-mount scanning, colons were washed with cold phosphate-buffered saline, whole-mounted, and fixed in 4% paraformaldehyde for 3 hours at room temperature. In situ hybridization, immunohistochemistry, multiplex, and whole-mount staining were completed using standard techniques described in detail in the Supplementary Methods.

### Organoid Culture

Organoid cultures were started and maintained principally as described by Sato et al,8 except that Noggin was replaced by 0.1 μg/mL recombinant GREM1 protein (R&D Systems, Minneapolis, MN). Culture techniques are described in detail in the Supplementary Methods.

### Imaging and Staining Quantifications

HALO software (Indica Labs, Albuquerque, NM) was used for quantification of *Grem1*-staining at ulcers. Regions were drawn demarcating the ulcer bed and the first 0.5 mm of colon tissue on either side of the ulcer, the latter further separated into muscularis propria, muscularis mucosa, and lamina propria. *Grem1* staining positivity was measured as the percentage area of each region. Quantifications of other staining were done using Qupath open-source digital software (https://qupath.github.io/). Annotation objects demarcating ulcer-adjacent crypts and wound-associated epithelium (WAE) were drawn, and cells were automatically detected by Qupath. The nuclear or cellular mean 3,3’-diaminobenzidine tetra hydrochloride optical densities (mDOPs) of all cells were normalized by subtracting the lowest detected nuclear or cellular mDOP in that image. Cells with normalized mDOPs above a set threshold per staining target were counted as positive. For Alcian blue staining quantification, “hematoxylin” values were set to red color values and “stain 2” to blue color values to adapt to the different chromogens used. The counting of positive crypts in en face sections of DSS-treated animals was done manually, aided by automatic positivity labeling of individual cells by Qupath.

For *Bmp4* in situ hybridization (ISH) quantification, extra care was taken to discriminate between an epithelial and stromal signal source, and any epithelial cells with ≥2 subcellular 3,3′-diaminobenzidine tetra hydrochloride spots were regarded positive, while for tandem dimer (td)Tomato immunohistochemistry (IHC) quantification, any cells with nuclear mDOP (nmDOP) >0.1 were regarded positive. A crypt with more than half of its cells being positive were counted as positive crypts. The counting of fully clonal crypts at steady-state conditions was done manually. The fraction of traced cells per cell position in the crypt and the number of traced cells per villus were manually quantified independently by 3 different observers, and their values were averaged. For quantifying fluorescence images, the mean cellular pixel values of all detected cells were normalized per channel by subtracting the lowest detected mean cellular pixel value. Cells with a normalized mean cellular pixel value above a set threshold per channel were counted as positive. Quantification of costainings of tdTomato IHC or *Bmp4* ISH with Ki67 or differentiation markers was done manually in Qupath. Detection of overlap with the *Grem1* ISH staining pattern was done using the “Colocalization Threshold” option in ImageJ software (National Institutes of Health, Bethesda, MD). Quantification of organoid area was performed using the plugins “Adaptive Thr” and “MorphoLibJ” in ImageJ.

### Colon Ulcer Measurements

Ulcer width was measured as the distance between the 2 ulcer-adjacent crypts, following the curve of the muscularis mucosa, if present. WAE length was measured as the length of the epithelium covering the ulcer bed until its transition into the ulcer-adjoining crypt. The degree of ulceration was determined by measuring the total length of denuded regions over the total distal colon length, from the proximal folds down.

## Results

### *Panepithelial Expression of* Bmp4 *Reduces Crypt Base Columnar* Lgr5 *Expression*

**Figure 1:**
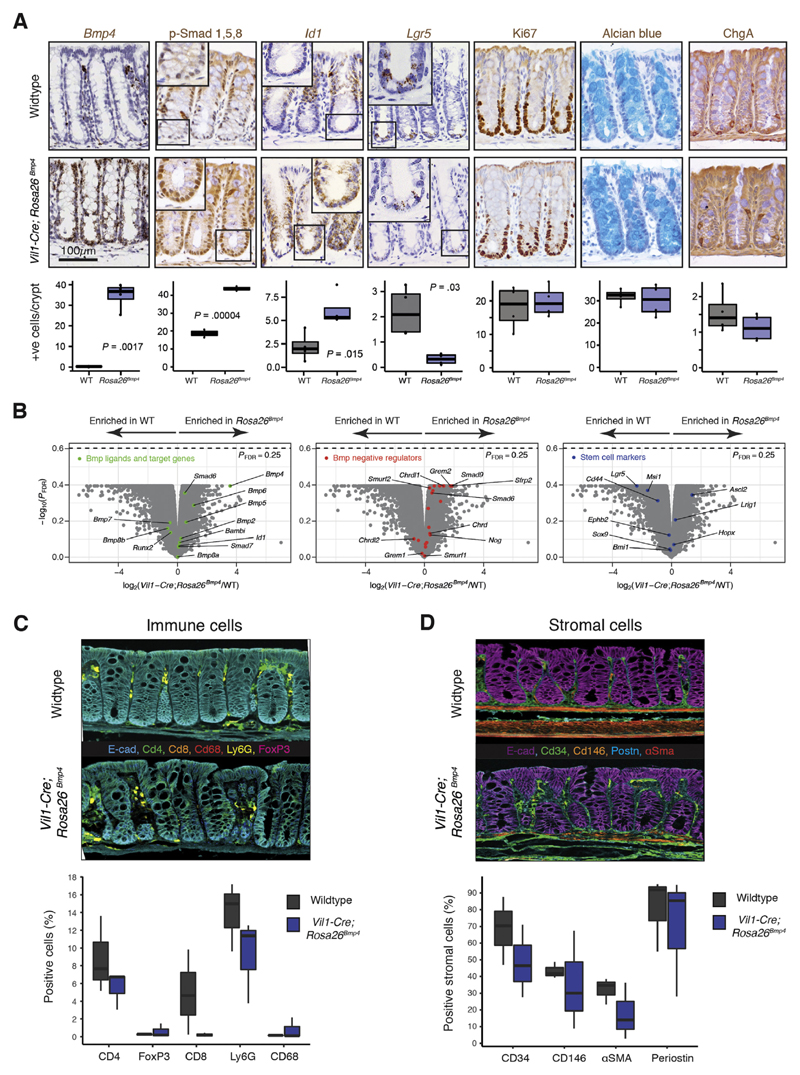
Steady-state *Vil1-Cre*;*Rosa26*^*Bmp4*^ mouse colonic phenotyping. (*A*) ISH/IHC phenotyping and cell quantification of colon in steady-state *Vil1-Cre*;*Rosa26*^*Bmp4*^ and wild-type (WT) control mice (*t* test; n = 4 mice per genotype). *Inset* magnification 400×. p-, phosphorylated. +ve, positive. (*B*) Volcano plots show differential gene expression between WT and *Vil1-Cre*;*Rosa26*^*Bmp4*^ animals (n = 3 mice per genotype). Multiplex IHC and cell quantification show (*C*) colonic immune and (*D*) stromal cell landscapes in WT and *Vil1-Cre*;*Rosa26*^*Bmp4*^ animals (n = 3 per genotype). Box-and-whisker plots: The *horizontal line* in the middle of each *box* indicates the median; the *top and bottom borders* of the box mark the 75th and 25th percentiles, respectively, the *whiskers* mark minimum and maximum of all the data, and the *circles* indicate outliers. E-cad, E-cadherin; FDR, false discovery rate.

To assess the effect of BMP ligand expression on epithelial cell fate, we generated a conditional *Bmp4*-expressing mouse (*Rosa26^Bmp4^*) and crossed this with an epithelial-specific *Cre* recombinase to drive *Bmp4* ligand expression along the intestinal vertical axis (*Vil1-Cre*;*Rosa26^Bmp4^*) (Supplementary Figure 1A). This abrogated the physiological gradient of BMP target gene expression in the epithelium, with *Id1* and phosphorylated Smad1, 5, 8, expression seen extending into the crypt bases (Figure 1A). Molecular and morphologic phenotyping of the animals showed a reduction in intestinal *Lgr5* expression and crypt base columnar (CBC) cell number in the epithelium^17^ (Figure 1A and B). There was subtle variation in the stromal and immune cell landscapes between mice, but this did not reach statistical significance on cell quantification, except for fewer macrophages and Cd8 cells in *Vil1-Cre*;*Rosa26^Bmp4^* small intestine (Figure 1C, Supplementary Figure 1). Notably there was no overt pathologic phenotype developing in steady-state mice up to 455 days after recombination, indicating homeostatic compensation, which may be mediated by the enrichment of an array of BMP antagonists and signal transduction negative regulators in the *Vil1-Cre*;*Rosa26^Bmp4^* model (Figure 1B).

### *Epithelial* Bmp4 *Ligand Expression Alters Individual Secretory Cell Fate Determination*

**Figure 2:**
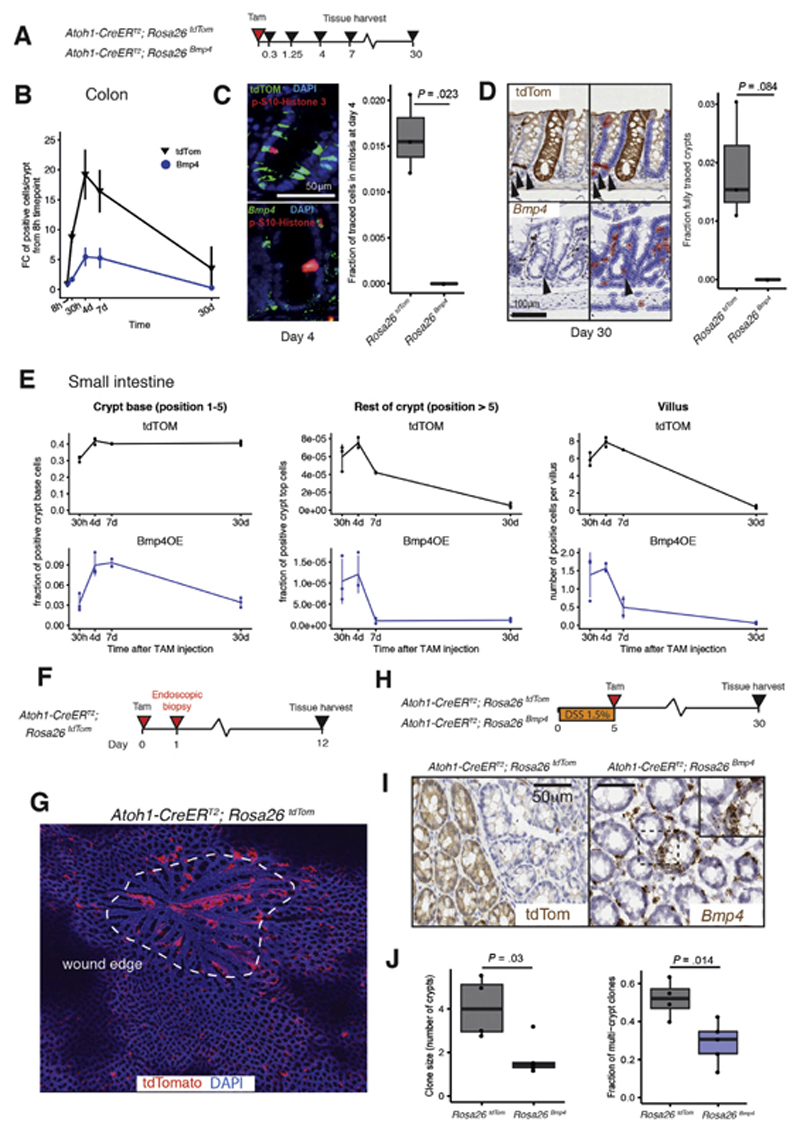
BMP ligand exposure impacts secretory progenitor cell fate. (*A*) Schematic shows recombination and harvesting of homeostatic secretory cell mouse models. (*B*) Number of colonic epithelial cells stained with tdTomato IHC (*black lines*) or *Bmp4* ISH (*blue lines*) over time after recombination. *P* < .001 for genotype, time point, and genotype: time point interaction effects from a 2-way analysis of variance. *Statistical significance of comparisons between tdTomato and *Bmp4* at each time point from Tukey’s Honest Significant Difference post hoc tests (n = 3 mice per group, 7 days: n = 2 mice per genotype). (*C*) Costain of phosphorylated (p)-histone3 (*red*) with tdTomato IHC (*top panel, green*) or *Bmp4* ISH (*bottom panel, green*). Quantification of fraction of tdTomato- or Bmp4-expressing colonic cells undergoing cell proliferation at day 4 after recombination (t test; n = 3 mice per genotype). (*D*) Long-lived secretory cells and lineage-traced crypts 30 days after recombination, stained with tdTomato IHC or *Bmp4* ISH and automated detection of positive cells with a digital pathology platform used to exclude noncontributory stromal cell staining (QuPath). Quantification of fraction of fully traced crypts in different genotypes at day 30 (t test, n = 3 mice per genotype). *P* = .084 with no fully Bmp4-traced crypts detected. *Arrowheads:* epithelial cells marked as positive. (*E*) Fraction or number of tdTomato IHC- or *Bmp4* ISH-stained cells in the small intestinal crypt base, rest of crypt, and on the villus over time after recombination (n = 3 mice per group, 7 days: n = 2 mice per genotype, 30 crypts and villi analyzed per mouse) (*F*) Schematic shows recombination and harvesting of endoscopy biopsy-wounded secretory cell mouse model. (*G*) En face section of healing endoscopic biopsy wound at day 12 (*dashed white line*) in *Atoh1-CreER^T2^*;*Rosa26^tdTom^* animals shows streaming of recombined cells into the wound bed. (*H*) Schematic shows recombination and harvesting of DSS-treated secretory cell mouse models. (*I*) Representative en face sections of colon from secretory cell models 30 days after initiation of DSS treatment show epithelial cell expression of tdTomato or *Bmp4.* (*J*) Clonal patch quantification shows an increase in multicrypt patch size and number in *Atoh1-CreER^T2^*;*Rosa26^tdTom^* animals after DSS treatment (t test, n = 5 *Rosa26^Bmp4^* mice, 4 *Rosa26^tdTom^* mice). The *error bars* represent the standard deviation. Box-and-whisker plots: The *horizontal line* in the middle of each *box* indicates the median; the *top and bottom borders* of the box mark the 75th and 25th percentiles, respectively, the *whiskers* mark minimum and maximum of all the data, and the *circles* indicate outliers. DAPI, 4′,6-diamidino-2-phenylindole. FC, fold change; TAM, tamoxifen; *P < 0.05, ** *P* < 0.01.

Beumer et al^18^ showed that BMP ligands regulate enteroendocrine cell fate along the crypt-villus axis, so we assessed the effect of autocrine expression of *Bmp4* on the fate of individual secretory progenitor cells. To do this, we used *Atoh1-CreER^T2^* to induce expression of tdTomato marker (*Atoh1-CreER^T2^*;*Rosa26^tdTom^*) or *Bmp4* (*Atoh1-CreER^T2^*;*Rosa26^Bmp4^*) in individual *Atoh1* -positive cells in steady-state conditions. We then spatiotemporally tracked cells expressing the active morphogen *Bmp4* (with ISH) or the functionally neutral tdTomato marker (with IHC), taking care to quantify and contrast the spatiotemporal fate of cells within, and not directly between animal groups, by using these different methodologic cell-marking techniques (Figure 2A).

In the colon, discrete secretory progenitors expressing tdTomato or *Bmp4* were identified 8 hours after recombination (Figure 2B, Supplementary Figure 2A). Cells marked with tdTomato actively proliferated (Figure 2C). The marked population peaked at day 4, with expression seen in most of the goblet cells throughout the colonic crypts. Thereafter, the marked cell population declined, consistent with terminal differentiation, but ongoing tdTomato expression at 30 days was seen in a population of persistent colonic secretory cells as well as a proportion of fully lineage-traced crypts (Figure 2D). In *Atoh1-CreER^T2^*;*Rosa26^Bmp4^* animals, there was an absence of cell proliferation in cells expressing functionally active *Bmp4*, resulting in profound reduction in *Bmp4*-marked cells at all subsequent times (Figure 2B). At 30 days, there were very few long-lived *Bmp4*-expressing cells, and no fully traced crypts were seen (Figure 2D), indicating abrogation of the homeostatic secretory cell lineage tracing seen in occasional individual crypts of wild-type mice.

To assess the impact of BMP ligand on different secretory lineages, we mapped the cell position of tdTomato- or *Bmp4*-expressing epithelial cells in the small intestine over the same time frame (Supplementary Figure 2B). Consistent with previous findings,19 expression of the neutral tdTomato marker was seen in crypt basal cells within 30 hours and was retained in equivalent numbers of long-lived Paneth cells for 30 days (Figure 2E, Supplementary Figure 2B). In contrast, cells at the crypt base, marked by expression of the active morphogen *Bmp4*, reduced in number after day 7, with only one-third of labeled cells retained at 30 days. The proportion of *Bmp4*-expressing cells in the upper crypt and villus also declined more precipitously than cells expressing tdTomato (Figure 2E; Supplementary Figure 3A). Costaining of individual cell types showed skewed secretory cell determination at day 4 after wounding (Supplementary Figure 3B and C). These data show that in steady state, precocious exposure of secretory progenitors to the BMP ligand prevents cell proliferation, expedites terminal differentiation, reduces long-lived secretory cell survival, and inhibits cell dedifferentiation.

### Bmp4 *Expression in Secretory Progenitors Reduces Contribution to Regenerative Response*

Dedifferentiation of Atoh1-positive secretory progenitor cells is critical for the regenerative response to intestinal wounding.^19,20^ To illustrate this, we undertook endoscopic biopsy wounding of recombined *Atoh1-CreER^T2^*;*Rosa26^tdTom^* animals, collecting tissue at 12 days after wounding to assess discrete ulcer crypt dynamics (Figure 2F). Lineage-traced crypts were seen surrounding the ulcer bed, with columns of tdTomato-positive epithelial cells streaming into the wound center, indicating that secretory lineage cells contributed to colonic wound healing by secondary intention^21^ (Figure 2G). To assess the impact of *Bmp4* skewing of secretory cell fate on intestinal regeneration, we quantitatively assessed crypt clonal tracing in DSS-treated *Atoh1-CreER^T2^;Rosa26^tdTom^* and *Atoh1-CreER^T2^*;*Rosa26^Bmp4^* animals, using epithelial *Bmp4* or tdTomato marker expression in en face sections, 30 days after initiation19 (Figure 2H). In DSS-treated *Atoh1-CreER^T2^*;*Rosa26^Bmp4^* animals, there was a significant reduction in multiple crypt patch size and number in contrast to the extensive clonal expansions seen in similarly treated *Atoh1-CreER^T2^*;*Rosa26^tdTom^* mice (Figure 2J, Supplementary Figure 3D and E). This indicates that the skewing of the fate of individual secretory progenitor cells away from dedifferentiation reduces the contribution of *Bmp4*-expressing lineages to colonic regeneration.

### Mapping the Bone Morphogenetic Protein Signaling Landscape in Mouse and Human Colonic Regeneration

**Figure 3:**
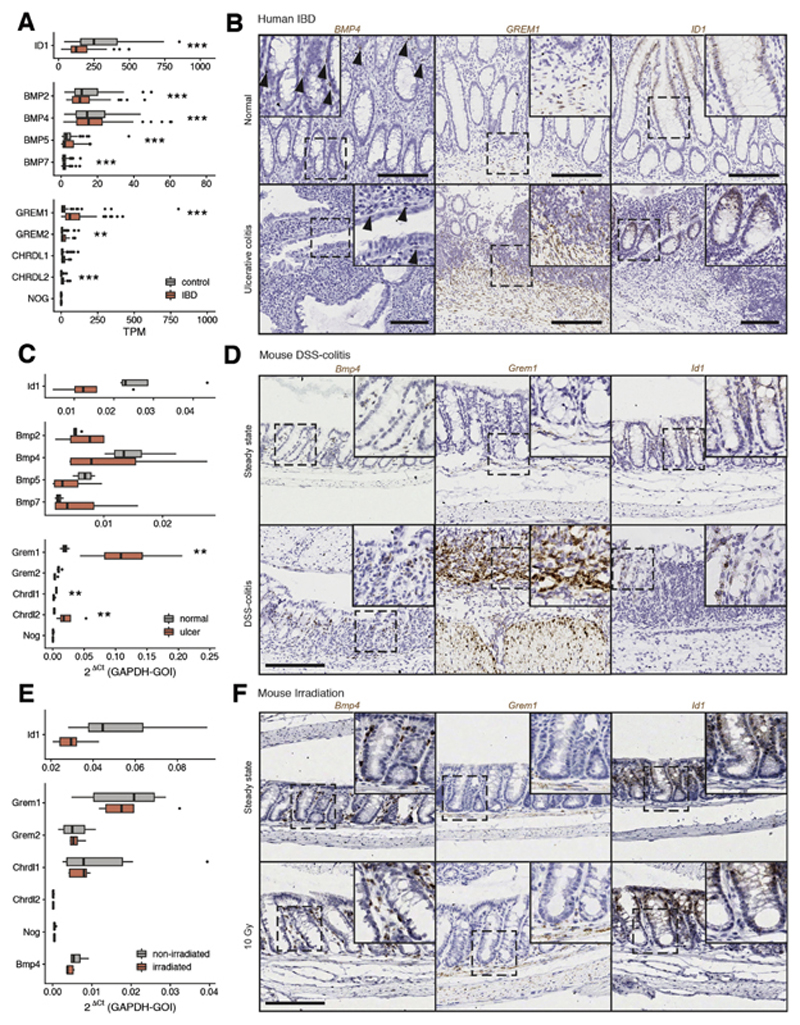
BMP signaling activity in human IBD and mouse models of intestinal regeneration. (*A*) Gene expression analysis of publicly available RNAseq data from human healthy and IBD tissue (GSE83687; n = 74 IBD, n = 60 control) shows downregulation of direct BMP target gene expression (ID1), variable impact on BMP ligand expression, and corresponding strong upregulation of *GREM1* as the key intestinal BMP antagonist. TPM, transcripts per million. (*B*) ISH of *BMP4, GREM1,* and *ID1* in healthy human colon and severe ulcerative colitis. (*C*) Colonic gene expression measured by quantitative reverse-transcription polymerase chain reaction (qRT-PCR) in mouse steady-state and DSS colitis shows downregulation of direct BMP target gene expression (Id1) with corresponding strong upregulation of *Grem1* (n = 4 mice; *P* = .029 by Mann-Whitney *U* test). (*D*) ISH hybridization of *Bmp4, Grem1,* and *Id1* in mouse steady-state and DSS colitis colon. (*E*) Colonic gene expression measured by qRT-PCR in mouse steady-state colon and 24 hours after 10-Gy whole-body irradiation shows minimal impact of radiation damage on BMP pathway constituent expression (n = 5 irradiated, 6 nonirradiated mice; no compared groups were significantly different). (*F*) ISH of *Bmp4, Grem1,* and *Id1* in mouse steady-state colon and after 10-Gy irradiation. Scale bars: 200 μm, magnification applies to all images, except insets. Box-and-whisker plot: The *horizontal line* in the middle of each *box* indicates the median; the *top and bottom borders* of the box mark the 75th and 25th percentiles, respectively, the *whiskers* mark the standard deviation, and the *circles* indicate outliers. GAPDH-GOI, ratio of glyceraldehyde 3-phosphate dehydrogenase gene to the gene of interest. Statistical differences were tested using empirical Bayes moderated 2-tailed *t* tests with false discovery rate correction (*A*) or Mann-Whitney tests with false discovery rate correction (*C* and *E*). **P < .01,*** *P* < .001.

Previous work in the colon and skin has suggested that physiological attenuation of the BMP pathway is required for regeneration,^22,23^ and this was consistent with our finding that autocrine ligand exposure impaired secretory cell dedifferentiation capacity. Next, we mapped the BMP signaling landscape in human IBD and 2 distinct mouse intestinal regeneration models (Supplementary Figure 4A and B). In human IBD, overall pathway activity, assessed by target gene expression, was significantly decreased in individuals with IBD. This correlated with a corresponding increase in the expression of a single intestinal secreted BMP antagonist, *GREM1* (Figure 3A). We confirmed these findings in IBD resection specimens, with upregulated *GREM1* expression seen arising from both the ulcer bed and the muscularis layers (Figure 3B, Supplementary Figure 4C).

Next, we assessed pathway activity in mouse DSS colitis models and after 10-Gy irradiation. The same pattern of disrupted BMP component expression was observed in murine DSS colitis and confirmed morphologically (Figure 3C and D). However, the nonulcerating epithelial cell injury provoked by intestinal irradiation did not provoke such significant BMP pathway dysregulation (Figure 3E and F; Supplementary Figure 4D and E). Together, these data show a comparable pattern of BMP pathway suppression after barrier breach in human and mouse, with correlative stromal upregulation of *GREM1* in human IBD and *Grem1* in mouse DSS colitis tissue.

### Topographically Distinct Stromal Cell Populations Are the Source of Increased Grem1 Expression

**Figure 4:**
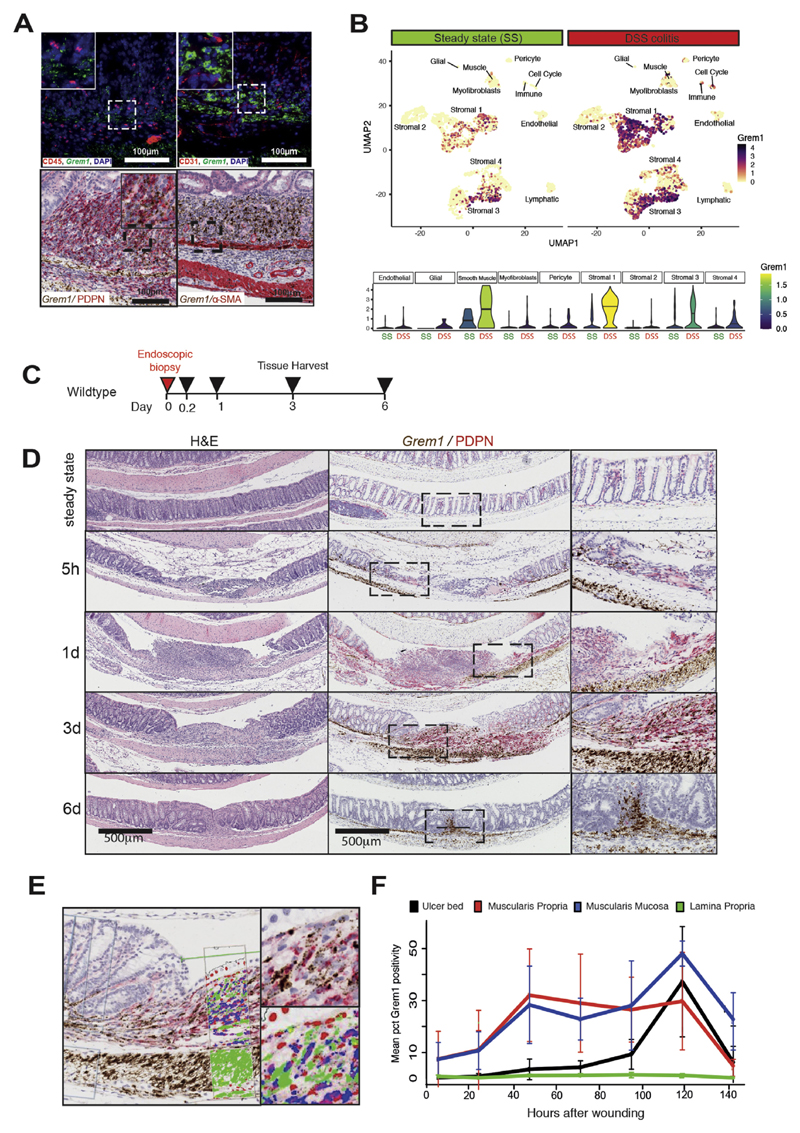
Two stromal cell populations upregulate *Grem1* in response to colonic injury. (*A*) ISH for *Grem1* expression with concomitant IHC costaining for stromal cell identification. There was no overlap of fluorescent expression of *Grem1* mRNA (*green*) with leucocytes marked by CD45 (*red*) or endothelial cells marked by CD31 (*red*). Overlapping chromogenic ISH for *Grem1* (*brown*) was seen with both ulcer bed stromal cells marked with podoplanin (PDPN) IHC (*red*), and muscularis mucosae/propria cells marked by αSMA IHC stain (*red*). DAPI, 4′,6-diamidino-2-phenylindole. (*B*) Small conditional RNA uniform manifold approximation and projection plots show upregulation and diversification of *Grem1* -expressing stromal cell populations in mouse steady-state (SS) and DSS colitis. Violin plots show *Grem1* expression in SS and DSS colitis in the mesenchymal cell subsets identified by Kinchen et al.10 Crossbars in Kinchen18 is median expression. (*C*) Schematic of biopsy schedule and tissue harvesting for endoscopic biopsy-wounded mice. (*D*) H&E and *Grem1* chromogenic ISH (*brown*) cos-tained with PDPN IHC (*red*) of biopsy-wounded wild-type mice at 5 hours, 1 day, 3 days, and 6 days after wounding. Scale bar: 500 μm. (*E*) Digital pathology false color markup of a Grem1/PDPN costaining image at 3 days after wounding. (*F*) Spatio-temporal quantification of *Grem1* -positivity in different colon tissue layers in and within 0.5 mm of the wound. The *error bars* represent the standard deviation. pct, percentage

Having identified profound upregulation of a single BMP antagonist in mouse DSS colitis models, we used a dual morphomolecular approach to characterize the *Grem1*-expressing cell population(s). First, we excluded *Grem1* expression arising from immune and vascular cells (Figure 4A, Supplementary Figure 5A and B). We then used dual ISH/IHC to spatially segregate *Grem1*-expressing mesenchymal cells into 2 topographically distinct groups: *α*-smooth muscle actin (SMA)-marked muscularis mucosae/propria cells and a previously demonstrated, inflammation-expanded, heterogenous population of stromal cells in the ulcer bed, broadly marked by podoplanin (PDPN/gp38),^24,25^ hereafter referred to as wound-associated stromal cells (WASCs) (Figure 4A). We used published stromal single cell RNA sequencing data from DSS colitis models,10 in combination with dual ISH/IHC, to assess the coexpression and spatial distribution of established and functionally relevant stromal subpopulations within these broader mesenchymal groups (Figure 4B, Supplementary Figure 5). We were able to find overlap of *Grem1* expression with some key morphogens and cytokines implicated in colonic regeneration, with stromal subsets coexpressing *Grem1* and *Rspo3,*^26^ *Ptgs2* (*cyclooxygenase-2*),^27^ and *Il33*.^28^ However, there was no significant overlapping expression with other stromal cells previously identified as subset markers^10^ or mesenchymal Wnt ligand sources, including *Foxl1,*^29^ *Gli1,*^30^ and *Wnt5a*^21^ (Supplementary Figure 5C–E).

DSS colitis is a simple and reproducible model for provoking a colonic regenerative response, but is not a pathogenic phenocopy of human IBD, and the response can be variable according to confounding factors such as the mouse microbiome and genetic background.31 It is not a suitable model for temporal interrogation, because ulceration initiation can occur at an unknown point during the 5- to 7-day administration period.

To assess the spatiotemporal dynamics of *Grem1* expression from stromal cells after injury, we undertook endoscopy-guided colon biopsy wounding (Figure 4C). Strikingly, *Grem1* expression was upregulated in the muscularis mucosa/propria of the ulcer environs as early as 5 hours after injury and persisted to at least day 6 after wounding (Figure 4D). Podoplanin cell staining was seen in the ulcer bed within 24 hours; however, very little *Grem1* expression was observed from WASCs until >48 hours after injury induction. These results indicated that increased expression of *Grem1* from the muscularis and WASC populations was both spatially and temporally distinct but had a cumulative effect of generating a rapidly induced and sustained increase in *Grem1* expression in the vicinity of the wound (Figure 4E and F).

### Genetic Manipulation of Bone Morphogenetic Protein Signaling Activity Affects Colonic Regenerative Capacity

**Figure 5:**
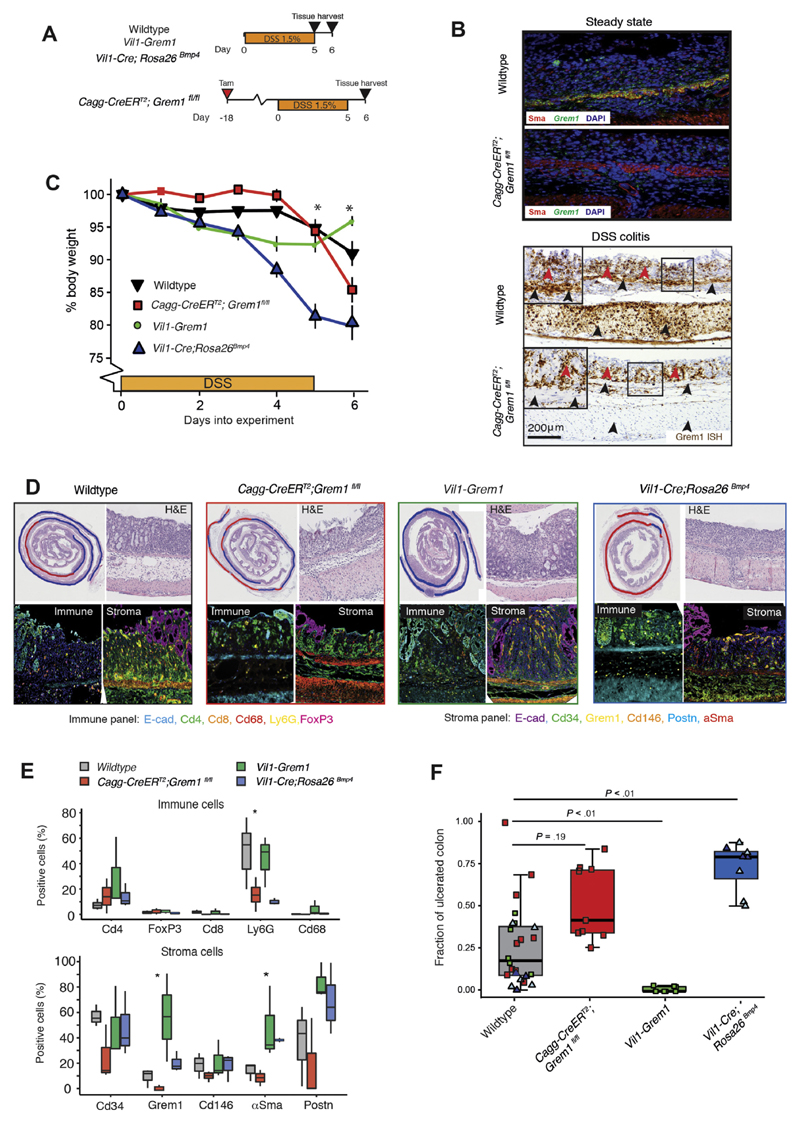
Functional impact of BMP manipulation on tissue regeneration. (*A*) Schematic of recombination (where applicable), DSS administration, and tissue harvesting schedule for constitutive and inducible mouse models. (*B*) In steady state, *Grem1* ISH (*green*) and αSMA IHC (*red*) show clear colocalization of expression in the muscularis mucosa of wild-type animals and efficient knockout of *Grem1* expression in *Cagg-CreER^T2^*;*Grem1^fl/fl^* animals. In DSS colitis, successful knockout of *Grem1* expression is seen from the tissue-resident muscularis mucosa and propria (*black arrowheads*), but continued *Grem1* expression (*brown stain*) from the wound-associated stromal cells persists (*red arrowheads*). DAPI, 4′,6-diamidino-2-phenylindole. (*C*) Body weight of wild-type, *Cagg-CreER^T2^*;*Grem1^fl/fl^*, *Vil1-Grem1,* and *Vil1-Cre*;*Rosa26^Bmp4^* mice over time as the percentage of body weight at the start of DSS treatment (n = 6-8 mice per genotype; 5 of 8 *Rosa26^Bmp4^* mice were killed at day 5 along with 5 of 8 wild-type littermates. *P < .05, indicating statistical significance from *t* tests comparing WT and *Vil1-Cre*;*Rosa26^Bmp4^* at each time point. The *error bars* represent the SEMD. (*D*) Representative H&E images of DSS-induced ulceration with *blue* and *red curves* demarcating the extent of normal and denuded colon, respectively. Multiplex IHC stain shows immune and stromal cell landscapes of DSS-induced ulcers in wild-type (*grey box*), *Cagg-CreER^T2^*;*Grem1^fl/fl^* (*red box*), *Vil1-Grem1* (*green box*), and *Vil1-Cre*;*Rosa26^Bmp4^* mice (*blue box*). (*E*) Cell quantification of multiplex IHC immune and stromal cell stain in DSS ulcers between genotypes (n = 3 per genotype). *P < .05 by analysis of variance. (*F*) Fraction of ulcerated colon in different animal genotypes. Control *colored dots* represent control littermates for the individual genotype experiments. The *light blue triangles* are 5 of 8 *Vil1-Cre*;*Rosa26^Bmp4^* animals that exceeded weight loss limits and had to be killed at 5 days; an equal number of wild-type littermates were killed as time point controls. The *dark blue triangles* are wild-type or *Vil1-Cre*;*Rosa26^Bmp4^* animals that reached the 6-day experimental end point (3 of 8) (n = 6-9 mice per genotype). The *horizontal line* in the middle of each *box* indicates the median; the *top and bottom borders* of the box mark the 75th and 25th percentiles, respectively, and the *whiskers* mark minimum and maximum of all the data. *P < 0.05, ** *P* < 0.01 by *t* test.

To assess the importance of secreted BMP antagonism in suppressing physiological signaling in DSS colitis, we used *Cagg-CreER^T2^*;*Grem1^fl/fl^* mice to knockout intestinal stromal *Grem1* expression^7,32^ (Figure 5A). Although efficient recombination was seen in the *α*SMA-positive muscularis cells throughout the colon, WASCs continued to robustly express *Grem1* in colonic ulcer beds (Figure 5B). Consequently, any potential detrimental effect of the conditional Grem1 knockout on wound healing fell below our significance threshold (Figure 5C, D, and F). Multiplex IHC showed no significant quantifiable variability in stromal cell marker expression in this mouse genotype (Figure 5D and E), so these data indicate that WASCs arise from activation of stromal cell populations that are not effectively recombined in *Cagg-CreER^T2^* animals.33 Concomitant expression from these populations can partially compensate for *Grem1* knockout in tissue-resident *α*SMA-positive muscularis cells.

This compensation arising from the heterogeneity of *Grem1* -expressing mesenchymal cells meant that to assess the functional effects and therapeutic potential of BMP pathway manipulation in regeneration, we needed to maximize agonism and antagonism of the pathway through epithelial overexpression of *Bmp4* (*Vil1-Cre*;*Rosa26^Bmp4^*) or *Grem1* (*Vil1-Grem1*),^7^ respectively (Figure 5A). Although *Vil1-Cre*;*Rosa26^Bmp4^* mice had no steady-state pathologic phenotype, these animals lost a significant amount of weight after DSS treatment, requiring early euthanasia in some cases (Figure 5C). Mice exhibited a dramatic phenotype, with complete distal colonic epithelial loss. In contrast, DSS treatment of *Vil1-Grem1* animals had minimal colitogenic impact, with barely detectable macroscopic ulceration (Figure 5D and F), although the colonic epithelium was hyperplastic.^7^ Together these data demonstrate that manipulation of the physiological mucosal BMP gradient, through epithelial ligand or antagonist expression, significantly alters intestinal regenerative capacity. An excess of BMP ligand exaggerates epithelial denudation in DSS colitis, whereas maximizing pathway antagonism through ectopic *Grem1* expression enhances wound healing at the expense of epithelial hyperplasia.

### Bone Morphogenetic Protein Antagonism and YAP/TAZ Signaling Convergently Impact Epithelial Cell Fate

**Figure 6:**
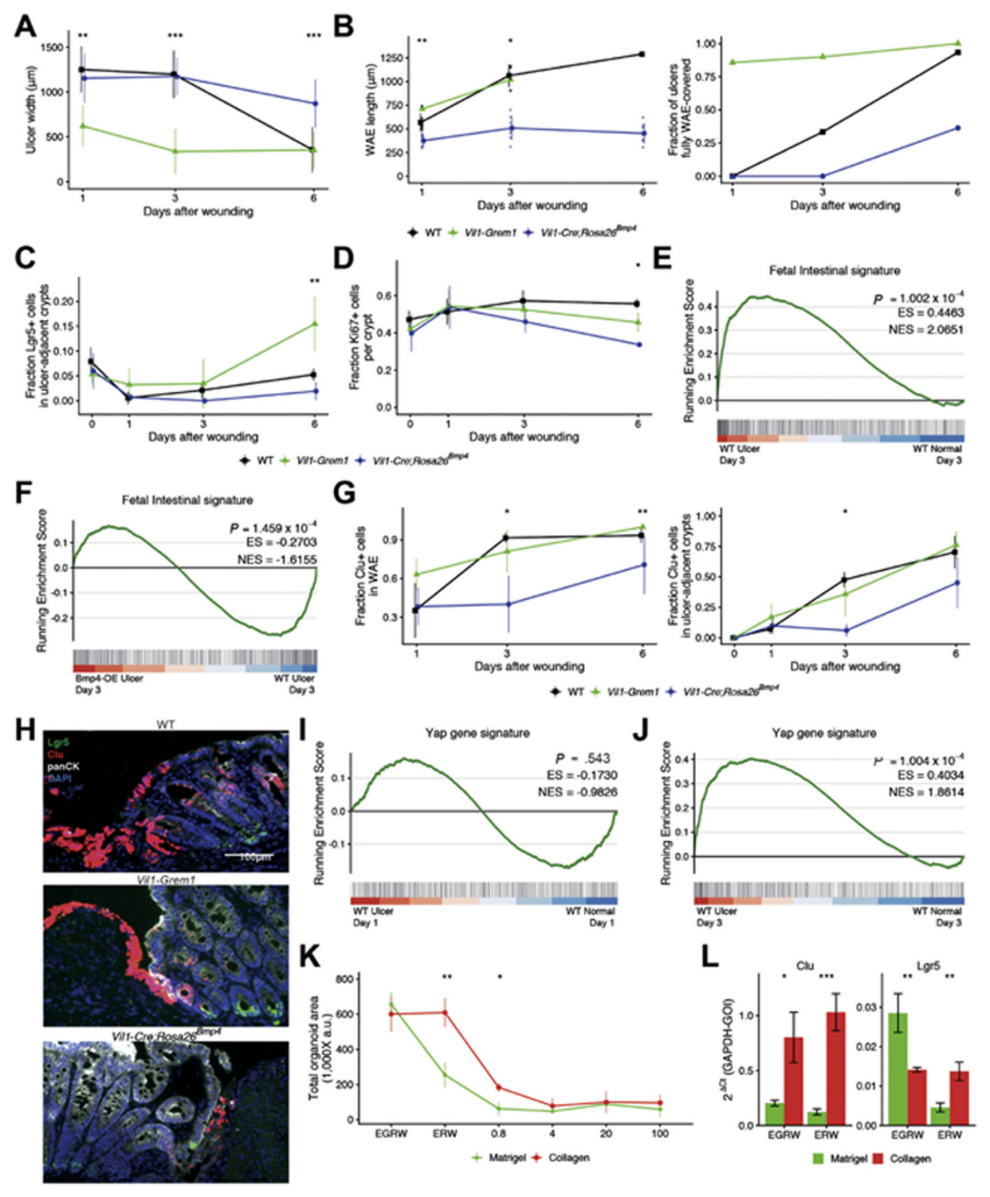
BMP manipulation affects epithelial adaptive response in wound healing. Microscopic appearance of ulcers in different genotypes over time after endoscopic wounding. (*A*) Ulcer width. (*B*) WAE length and the fraction of ulcer covered by WAE (n = 6-15 ulcers). Wounds completely covered with WAE were excluded from WAE length measurements. WT, wild-type. (*C*) Fraction of Lgr5-positive cells (n = 3–6 mice per group). (*D*) Ki67-positive proliferating cells in ulcer-adjacent crypts in different genotypes over time (n = 3–5 mice per group). (*E*) Gene set enrichment analysis showing enrichment of fetal intestinal signature in the endoscopy biopsy wound milieu at day 3. ES, enrichment score; NES, normalized enrichment score. (*F*) Negative enrichment of fetal intestinal signature in the biopsy wounds of *Vil1-Cre*;*Rosa26^Bmp4^* mice compared with wounded WT animals at day 3. (*G*) Regenerative stem cells, marked by clusterin (*Clu*) staining in (*left*) WAE and (*right*) ulcer-adjacent crypts in different genotypes, over time (n = 3-6 mice per group). In all *line plots,* line colors represent Wt (*black*), *Vil1-grem1* (*green*), and *Vil1-Cre*; *Rosa26^Bmp4^* (*blue*). (*H*) Representative images of ISH for *Lgr5* (green), *clusterin* (Clu; *red*) with costain IHC for pan-cytokeratin (panCK, *white*) and 4′,6-diamidino-2-phenylindole (DAPI, *blue*) in 3-day-old endoscopy wounds in different genotype animals. Scale bar: 100 μm. (I) Gene set enrichment analysis shows no enrichment of YAP signature in acute biopsy wounds in WT animals at day 1, but (*J*) significant enrichment of YAP signature in biopsy wound ulcers by day 3 as the wound bed remodels. (K) Organoid survival when grown in Matrigel (*green line*) or collagen (*red line*) with media containing variable recombinant proteins and increasing doses of recombinant BMP4 (n = 3 experiments, n = 2 for 20 ng/mL). (*L*) Quantitative reverse-transcription polymerase chain reaction of crypt base columnar (*Lgr5*) and regenerative stem cell gene expression (*Clu*) in organoids grown in Matrigel or collagen (n = 3 experiments) and with variable media constituents (*E*, epidermal growth factor; *G*, GREM1; *R*, RSPO1; *W*, WNT3A). GAPDH-GOI, ratio of glyceraldehyde 3-phosphate dehydrogenase gene to the gene of interest. The *error bars* represent the standard deviation. Statistical differences were tested using a Kruskal-Wallis test (*A–D*, *F*, *H*) or a Student *t* test (*I*). *P < .05, **P < .01, ***P < .001.

Because ulcer induction in the DSS model cannot be synchronized, we were unable to determine whether the effects of BMP pathway manipulation were the consequence of an altered injury or repair response. Therefore, we turned back to the endoscopic model, where the size of the biopsy forceps limited and controlled for the extent of initial ulceration, allowing us to assess the impact of manipulated BMP signaling on epithelial spatiotemporal dynamics in wound healing (Supplementary Figure 6A). Biopsy ulcers in *Vil1-Grem1* animals were rapidly covered by WAE (Figure 6A), whereas epithelial *Bmp4* expression in *Vil1-Cre*;*Rosa26^Bmp4^* mice delayed coverage by WAE and retarded closure (Figure 6B, Supplementary Figure 6B).

Next, we assessed stem and proliferating cell dynamics. Loss of CBC cells has been reported after intestinal injury,^34^ and Lgr5-expressing cell depletion was seen in ulcer-adjacent crypts immediately after wounding. CBC recovery from day 3 was enhanced by epithelial *Grem1* expression in the *Vil1-Grem1* model and delayed in *Vil1-Cre*;*Rosa26^Bmp4^* (Figure 6C, Supplementary Figure 6C). After an appropriate wound-induced spike in cell division in ulcer-adjacent crypts at 24 hours in all genotypes, proliferation slowed in *Vil1-Cre*;*Rosa26^Bmp4^* animals (Figure 6D, Supplementary Figure 6D), consistent with delayed reconstitution of CBC populations. We confirmed enrichment of fetal signatures^15^ in endoscopic biopsy wounds (Figure 6E) and showed that epithelial *Bmp4* ligand prevented expression of this reprogramming signature in 3-day-old wounds (Figure 6F).

Ayyaz et al^12^ showed that activation of Lgr5-negative regenerative stem cells was marked by expression of the molecular chaperone *çlusterin (Clu),* a gene that is also enriched in the fetal signature. We used multicolor ISH to track temporal expression of this cell marker over time after biopsy. In wild-type and *Vil1-Grem1* animals, *Clu* expression was rapidly activated in WAE and ulcer-adjacent crypts, and the *Clu*-positive cell population expanded over time. (Figure 6G and H, Supplementary Figure 6E). However, *Vil1-Cre*;*Rosa26^Bmp4^* animals exhibited a significant delay in *Clu* expression, which did not appear until after day 3 after injury. Together these data show that postwounding regenerative stem cell activation, marked by *Clu* expression and characterized by fetal gene signatures, was enhanced by BMP antagonism and temporally retarded by autocrine epithelial *Bmp4* signaling.

We noted that delayed but not entirely eliminated activation of Clu-positive regenerative stem cells after day 3 in *Vil1-Cre*;*Rosa26^Bmp4^* animal wounds occurred despite ongoing *Bmp4* expression. YAP/TAZ signaling plays a role in regulating adaptive reprogramming after barrier breach.^14,15^ We were able to confirm enrichment of YAP/TAZ signatures from mature endoscopic wounds at day 3 (Figure 6J) but saw no significant upregulation in biopsy wounds at day 1 (Figure 6I).

Given this temporal lag in YAP/TAZ activation, we postulated that matrix remodelling in mature wounds might independently contribute to delayed activation of regenerative (Clu-positive) stem cells in *Vil1-Cre*;*Rosa26^Bmp4^* animals. To assess this, we turned to organoid models, because growth in collagen matrix has been shown to simulate a remodeled wound bed by activating epithelial YAP/TAZ signaling and enriching for fetal signature expression.15 We cultured mouse intestinal organoids in Matrigel (Corning, Tewksbury, NY) and collagen and assessed the media BMP antagonist requirement (GREM1) and the tolerance of media BMP4 ligand in both conditions. Organoids grown in Matrigel were enriched for *Lgr5* expression, had an obligatory culture requirement for media BMP antagonist (GREM1) supplementation, and growth was inhibited by very low concentrations of BMP4 ligand (Figure 6K and L, Supplementary Figure 6F). In contrast, organoids grown in collagen were enriched for *Clu* over *Lgr5,* had acquired BMP antagonist independence, because they no longer required media GREM1 inclusion and were less sensitive to growth suppression by low doses of BMP4 ligand. Thus, organoid growth in collagen-enriched matrix can elicit a regenerative stem cell response through YAP/TAZ activation, and this abrogates the absolute requirement for BMP antagonism in organoid culture.

Altogether these results show that BMP manipulation in vivo can skew the dynamic temporal response to an acute wound, with exaggerated antagonism in the *Vil1-Grem1* model enhancing regenerative stem cell activation, CBC reconstitution, cell proliferation, and epithelial restitution. In contrast, epithelial *Bmp4* ligand overexpression delays and abrogates the epithelial reprogramming response, with impact on regenerative capacity. Although excessive BMP ligand delays the epithelial response, it does not entirely abrogate regenerative stem cell activation in remodeled wounds, and we show a temporally staggered but functionally convergent contribution from YAP/TAZ signaling to adapt epithelial cell fate across the duration of wound healing.

## Conclusions

Current IBD therapeutics reduce provoking inflammation^35^ with limited focus on enhancing the mucosal regenerative capacity because the mechanisms that regulate epithelial restitution are incompletely understood. Here, we have used a combination of models to map and functionally assess the impact of BMP pathway manipulation on epithelial cell fate in homeostasis and in the regenerative response to wounding. In steady state, autocrine epithelial *Bmp4* expression skews secretory cell fate, expediting terminal differentiation and inhibiting progenitor cell dedifferentiation. Because regeneration is underpinned by rapid epithelial adaptive cell reprogramming, there is a physiological requirement for attenuation of BMP signaling after injury. We show that this attenuation is mediated through upregulated expression of the secreted antagonist *Grem1* from topographically distinct stromal cell populations. Cumulative *Grem1* expression from independent mesenchymal cell populations contributes to rapidly initiated yet sustained BMP antagonism localized to the injury environs and permits capacity for functional compensation. Despite profound physiological stromal *Grem1* upregulation, we demonstrate functionally submaximal BMP antagonism after colonic ulceration, as ectopic epithelial *Grem1* expression in the *Vil1-Grem1* model markedly expedited colonic epithelial regeneration, highlighting the potential for BMP pathway manipulation in future IBD therapeutics. However, it is important to note that the enhancement of wound-healing capacity through permanent epithelial *Grem1* exposure comes at the longer-term expense of generating a hyperplastic and, ultimately, protumorigenic epithelial environment.^7^

The multicompartmental mucosal response to barrier breach is complex. Different studies have highlighted the involvement of other mechanisms in regulating intestinal regeneration, such as metabolic stress,^13^ mechano-transduction,^14,15^ and numerous other secreted cell-signaling pathways such as *TGFβ*^21^ and Wnt signaling.^21,26,36^ Here, we show variable requirement for BMP suppression in regulating gut regeneration after different modes of injury, with more pronounced disruption of mucosal BMP signaling provoked by ulceration than seen after intestinal irradiation. This reflects differences between the injurious stimuli, with irradiation predominantly damaging the epithelial cell compartment, while barrier breach invokes a multicompartmental tissue response

The use of spatiotemporal mapping to assess variability in cell signaling disruption after induction of intestinal injuries can help to triangulate the role of different pathways. Using this technique, alongside organoid models, we demonstrate a temporally staggered but functionally convergent effect of paracrine BMP antagonism and YAP/TAZ activation on promotion of the epithelial adaptive cell response in the ulcer milieu. Given the complexity of the multicompartmental response to wounding, it is likely that multiple different but frequently intersecting pathways act in a convergent fashion to alter epithelial cell fate determination. Understanding this synergism and any functional redundancy in the system will be vital for development of drugs that therapeutically manipulate the intestinal regenerative response.

The intestinal mucosa has evolved to generate an effective and rapid response to injury through temporary relaxation of stringent homeostatic cell fate control. Epithelial denudation, stromal activation, and immune cell infiltration induce transient disruption of physiological, polarized signaling gradients, which drives surrounding epithelial cell dedifferentiation and adaptive reprogramming. As wounds heal, barrier restitution, generation of new crypt architecture, and dissolution of immune and activated stromal cell infiltrate allow reimposition of graduated cell signaling and re-establish regimented cell fate determination along the crypt axis. Here we show that excessive BMP ligand exposure affects epithelial cell dedifferentiation capacity; hence, intestinal regeneration is dependent on physiological attenuation of this homeostatic signal, which occurs through time-limited upregulation of the secreted antagonist, GREM1. As we have previously shown, permanent overexpression of the same antagonist is protumorigenic in humans^37^ and mice,^7^ and this illustrates the fine line between dynamic, temporary disruption of signaling networks to physiologically adapt epithelial cell fate in regeneration and the pathologic cooption and corruption of the same pathways in neoplasia.

## Supplementary Material

Supplementary Material

## What You Need to Know

### Background and Context

Intestinal regeneration is underpinned by dedifferentiation and stem cell plasticity, but the pathways that regulate this profound adaptive cell reprogramming response are not understood.

### New Findings

Bone morphogenetic protein (BMP) pathway attenuation is required for epithelial dedifferentiation and intestinal regeneration and is physiologically mediated by upregulation of the secreted antagonist, Grem1, from topographically distinct populations of stromal cells. BMp pathway manipulation showed that antagonist-mediated BMP attenuation was obligatory, but functionally submaximal, because regeneration was impaired or enhanced by epithelial overexpression of *Bmp4* or *Grem1,* respectively.

### Limitations

Stromal cell heterogeneity prevented complete knockout of endogenous *Grem1* expression.

### Impact

Intestinal regeneration can be expedited by enhanced antagonism of the BMP pathway, highlighting the potential for therapeutic pathway manipulation in conditions such as inflammatory bowel disease.

## Abbreviations

BMP: bone morphogenetic protein
CBC: crypt base columnar
DSS: dextran sodium sulfate
IBD: inflammatory bowel disease
IHC: immunohistochemistry
ISH: in situ hybridization
mDOPs: mean 3,3′-diaminobenzidine tetra hydrochloride optical densities
RNAseq: RNA sequencing
SMA: smooth muscle actin
td: tandem dimer
WAE: wound-associated epithelium
WASC: wound-associated stromal cell
