# Supplementary material for: Bone Morphogenetic Protein Pathway Antagonism by Grem1 Regulates Epithelial Cell Fate in Intestinal Regeneration
Source: Gastroenterology. Author manuscript; Available in PMC 2022 Oct 23. (PMC7613733; doi:10.1053/j.gastro.2021.03.052)
Supplement: Supplementary Material [file EMS155869-supplement-Supplementary_Material.pdf]

Supplementary Methods

RNA Extraction and Gene Expression Analysis

For gene expression analysis by quantitative polymerase chain reaction (PCR), tissues and cells were lysed, and RNA was isolated using the RNeasy Mini kit (Qiagen, Valencia, CA). Organoids were first extracted from collagen matrix by incubating with collagenase VIII (0.6 μg/μL in medium; Sigma-Aldrich) for 5 minutes or from Matrigel by washing with ice-cold medium, before lysis. Then, 1 cm of distal colon (irradiation experiment) was homogenized using a handheld homogenizer (Polytron; Kinematica, New York, NY). RNA was treated with deoxyribonuclease (DNase) I (Thermo Fisher Scientific). Complementary DNA (cDNA) was generated using the High Capacity cDNA Reverse Transcription Kit (Thermo Fisher Scientific). To roughly normalize samples within an experiment, the same amount of RNA was taken per sample to make cDNA. Quantitative reverse transcription-PCR (qRT-PCR) was performed on the Applied Biosystems QuantStudio 6 Flex Real-Time PCR System using Fast Universal PCR Master Mix and TaqMan Gene Expression assays (Applied Biosystems, Foster City, CA), listed in Supplementary Table 1.

RNA Sequencing

Total RNA was extracted using the RNeasy Mini Kit (Qiagen) with on-column DNase treatment. RNA concentrations and purity were assessed using a NanoDrop One (Thermo Fisher). Samples with A260/A280 ratio <2 underwent further DNase treatment using DNase I, Amplification Grade (18068015, Thermo Fisher). RNA concentrations were measured using the Qubit RNA HS Assay Kit (Q32855, Thermo Fisher). RNA quality was assessed on a 2100 Bioanalyser Instrument (Agilent, Santa Clara, CA). Library construction and sequencing were performed at the Oxford Genomics Centre. Total RNA quantity and integrity were assessed using the Quant-IT RiboGreen RNA Assay Kit (Invitrogen, Carlsbad, CA) and the Agilent Tapestation. Messenger RNA purification, double-stranded cDNA generation, and library construction were performed using NEBNext Poly(A) mRNA Magnetic Isolation Module (E7490; New England Biolabs, Ipswich, MA) and NEBNext Ultra II Directional RNA Library Prep Kit for Illumina (E7760L; New England Biolabs) with in-house adapters and barcode tags (using dual indexing). The concentrations used to generate the multiplex pool were determined by a Quant-iT PicoGreen dsDNA Assay (P7589; Invitrogen). The final size distribution of the pool was determined using a TapeStation system (Agilent), and quantified using a Qubit assay (Thermo Fisher). Libraries were sequenced on the NovaSeq 6000 (Illumina) as 150-base pair paired-end reads.

In Situ Hybridization

Standard Advanced Cell Diagnostics RNAscope protocols (Advanced Cell Diagnostics Newark, CA) were followed for both chromogenic ISH (RNAscope 2.5 HD Assay) and fluorescent ISH (RNAscope Multiplex Fluorescent Assay v2). For the latter, fluorophores (Cy3 and Cy5) were diluted at 1:1500 in tyramide signal amplification buffer. The following Advanced Cell Diagnostics probes were used: Hs-*BMP4* (454301), Hs-*GREM1* (312831), Hs-*ID1* (414351), Mm-*Clu* (427891-C3), Mm-*Grem1* (314741), Mm-*Id1* (312221), Mm-*Rspo3* (402011), Mm-*Wnt5a* (316791), Mm-*Foxl1* (407401), Mm-*Gli1* (311001), Mm-*Il33* (400590), Mm-*Lgr5* (312171). Chromogenic IHC after ISH was started right after the 3,3′-diaminobenzidine tetra hydrochloride (DAB) washing step in the 2.5 high-definition assay protocol, and fluorescent IHC after ISH was started right after the last horseradish peroxidase-blocking step in the version 2 multiplex fluorescent assay protocol. For both staining types, IHC was started with 2× 5-minute washes in phosphate-buffered saline (PBS), followed by 1 hour of blocking. Antibodies were incubated overnight at room temperature or 4°C.

Immunohistochemistry Analysis

Sections were deparaffinized in xylene and rehydrated through graded alcohols to water. Antigen retrieval was done by pressure cooking in 10 mmol/L citrate buffer (pH 6.0) for 5 minutes for Ki67, followed by 2 minutes incubation in 0.5% Triton-X100 in PBS. Endogenous peroxidase activity was blocked by incubating in 3% hydrogen peroxidase (in water) for 30 minutes (for TdTomato staining, hydrogen peroxidase was diluted in methanol instead of water).

Next, sections were blocked with 5% serum for 1 hour, after which they were incubated with primary antibodies. Antibodies against the following proteins were used: PTGS2 (1:50, 610203; BD Biosciences, San Jose, CA), PDPN (1:500, 50256-RP02-50; Sino Biologicals, Beijing, China), αSMA (1:1000, A2547; Sigma-Aldrich), pan-cytokeratin (1:50, 3-9003-82; Thermo Fisher), phosphorylated Smad1/5/8 (1:200, AB3848-I; Merck), Ki67 (1:500, CS12202S; Cell Signaling Technology, Danvers, MA), chromogranin A (1:400, ab15160; Abcam, Cambridge, MA), lysozyme (1:500, EC3.2.1.17; DAKO, Glostrup, Denmark), LY6G (1:50, 551459; BD Biosciences), CD45 (1:300, 103127; BioLegend, San Diego, CA), F4/80 (1:50, 14-4801-82; Thermo Fisher,), CD31 (1:100; 77699S; Cell Signaling Technology), phosphorylated histone 3 (1:100, ab5176; Abcam), and tdTomato (1:500, NBP1-96752; Novus Biologicals, Centennial, CO). The sections were then incubated with appropriate secondary antibodies for 30 minutes at room temperature.

For chromogenic visualization, sections were incubated with avidin-biotin complex (ABC; Vector Laboratories, Burlingame, CA) for 30 minutes and stained using DAB solution (Vector Laboratories), after which they were counterstained with hematoxylin, dehydrated, and mounted. For IHC staining after chromogenic ISH, the ImmPRESS-AP polymer reagent and ImmPACT Vector Red AP kit (Vector Laboratories) were used to develop a red signal. In case fluorescent secondary antibodies were used, sections were incubated with 4′,6-diamidino-2-phenylindole (DAPI; RNAscope Multiplex Fluorescent kit v2) for 1 minute, then

mounted with ProLong Gold antifade mountant (Thermo Fisher). For other fluorescent visualizations, Tyramide SuperBoost kits (Alexa Fluor 594 or 647, Thermo Fisher) were used. Alcian blue staining was performed using the standard protocol. Briefly, slides were incubated in Alcian blue for 30 minutes and washed in water. They were then incubated in nuclear fast red solution (Sigma-Aldrich) for 5 minutes and washed in running tap water for 1 minute, followed by dehydration and mounting.

Multiplex Panel

Multiplex (MP) immunofluorescence (IF) staining was performed on 4-μm-thick formalin-fixed paraffin embedded (FFPE) sections using the OPAL protocol (Akoya Biosciences, Marlborough, MA) on the Leica BOND RX^m autostainer (Leica Microsystems, Wetzlar, Germany). Six consecutive staining cycles were performed using the following primary antibody-Opal fluorophore pairings:

Stroma panel: (1) Gremlin 1 (1:750, AF956; R&D)–Opal 540; (2) CD34 (1:3000, ab81289; Abcam)–Opal 520; (3) CD146 (1:500, ab75769; Abcam)–Opal 570; (4) SMA (1:1000, ab5694; Abcam)–Opal 620; (5) Periostin (1:1000, ab227049; Abcam)–Opal 690; and (6) E-cadherin (1:500, 3195; Cell Signaling)–Opal 650.

Immune panel: (1) Ly6G (1:300, 551459; BD Pharmingen)–Opal 540; (2) CD4 (1:500, ab183685; Abcam)–Opal 520; (3) CD8 (1:800, 98941; Cell Signaling)–Opal 570; (4) CD68 (1:1200, ab125212; Abcam,)–Opal 620; (5) FoxP3 (1:400, 126553; Cell Signaling)–Opal 650; and (6) E-cadherin (1:500, 3195; Cell Signaling)–Opal 690.

Primary antibodies were incubated for 60 minutes and detected using the BOND Polymer Refine Detection System (DS9800; Leica Biosystems, Buffalo Grove, IL) according to the manufacturer's instructions, substituting DAB for the Opal fluorophores, with a 10-minute incubation time and without the hematoxylin step. Antigen retrieval at 100oC for 20 minutes, in accordance with standard Leica protocol, with Epitope Retrieval (ER) Solution 1 or 2 was performed before each primary antibody was applied. Sections were then incubated for 10 minutes with spectral DAPI (FP1490, Akoya Biosciences) and the slides mounted with VECTASHIELD Vibrance Antifade Mounting Medium (H-1700-10; Vector Laboratories). Whole-slide scans and multispectral images (MSI) were obtained on the Akoya Biosciences Vectra Polaris. Batch analysis of the MSIs from each case was performed with the inForm 2.4.8 software provided. Finally, batched analyzed MSIs were fused in HALO (Indica Labs) to produce a spectrally unmixed reconstructed whole-tissue image, ready for analysis.

Whole-Mount Antibody Staining

Colon was sectioned into 2- to 3-cm pieces, washed in 0.1% PBS-Tween for 2 days, blocked in 10% donkey serum in PBS overnight at 4°C, and incubated with DAPI (10 μg/mL stock) in 10% donkey serum in PBS for 3 days. The tissue was finally washed with PBS-Tween for 1 day before being imaged on a TCS SP5 confocal microscope (Leica)

Organoid Culture

The culture medium to maintain the organoids was Advanced Dulbecco's Modified Eagle Medium/F12 (Thermo Fisher) supplemented with penicillin/streptomycin, 10 mmol/L HEPES, 1× Glutamax, 1× B27 supplement (Thermo Fisher), 1.25 mmol/L *N*-acetyl-L-cysteine (Sigma-Aldrich), 50 ng/mL recombinant murine epidermal growth factor (Thermo Fisher), RSPO1, WNT3A (EGF-RSPO1-WNT3A medium or ERW medium) and GREM1. The RSPO1 source was 10% conditioned medium. The WNT3A source was 50% conditioned medium from L cells (ATCCCRL-2647) prepared following the company's protocol.

Small intestinal organoids isolated from steady-state wild-type mice were mechanically chopped into small pieces using a small-orifice Pasteur's pipette and seeded in Matrigel (Corning) or collagen matrix. Collagen matrix was prepared on ice by adding 10× PBS (to make 1× PBS) and 1N NaOH (to make 25 mmol/L NaOH) to collagen I (rat tail; Thermo Fisher). Organoids were treated with ERW medium, ERW medium with GREM1, or ERW medium with different concentrations of recombinant mouse Bmp4 protein (R&D Systems). The organoids received new medium after 2 days and were imaged and harvested for RNA analysis after 4 days of treatment.

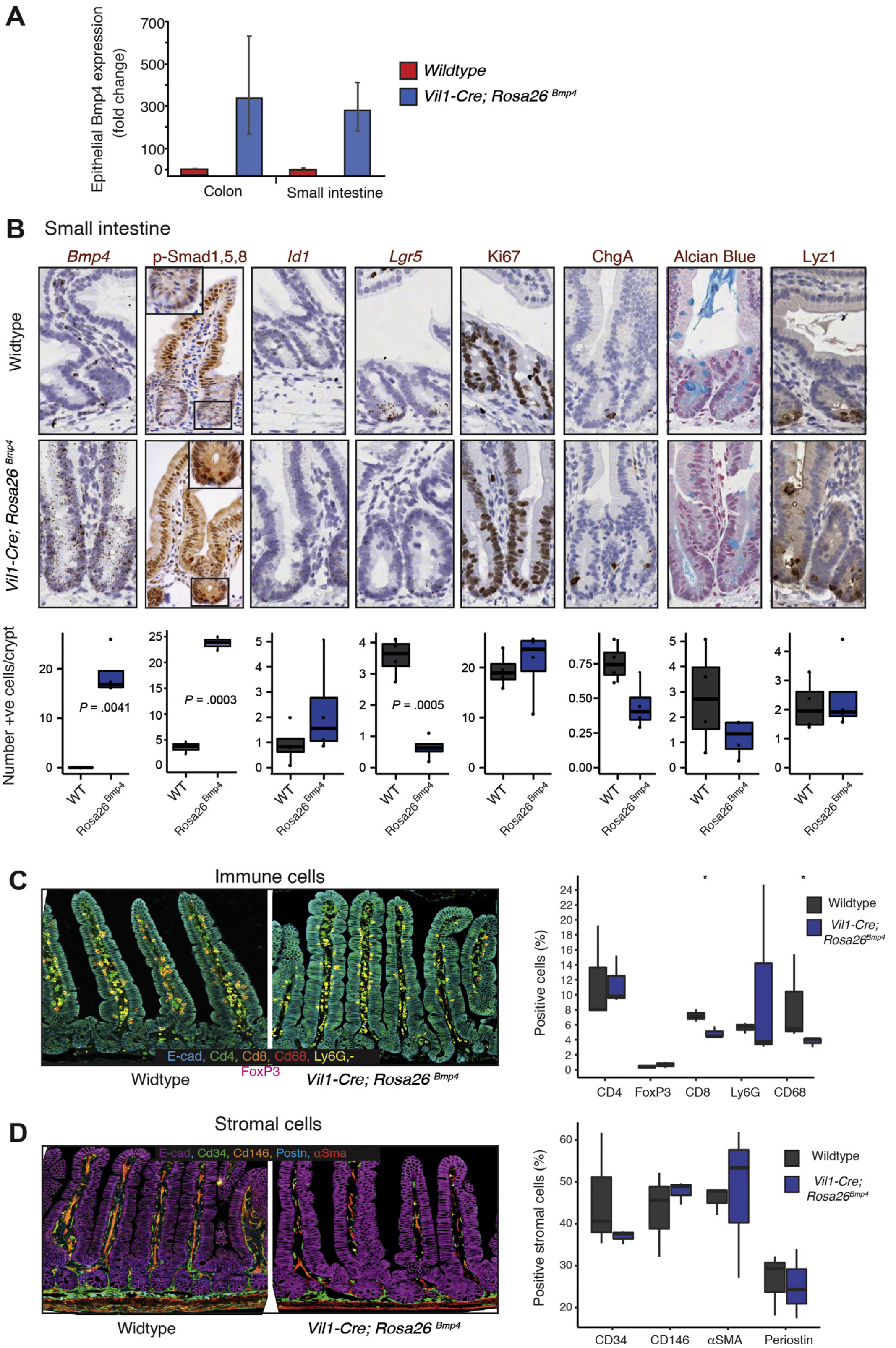

Supplementary Figure 1. Steady-state *Vil1-Cre*;*Rosa26*^{Bmp4} mouse small intestinal phenotype. (*A*) Epithelial expression of *Bmp4* (fold change) in wild-type and *Vil1-Cre*;*Rosa26*^{Bmp4} mice by quantitative reverse transcription polymerase chain reaction (n = 2 per genotype). The *error bars* show the standard deviation. $P < .05$ by *t* test. (*B*) ISH/IHC phenotyping and cell quantification of small intestine in steady state *Vil1-Cre*;*Rosa26*^{Bmp4} and wild-type (WT) control mice (n = 4 mice per genotype; *t* test). p-phosphorylated. Multiplex IHC and cell quantification to show small intestinal (*C*) immune and (*D*) stromal cell landscapes in wild-type and *Vil1-Cre*;*Rosa26*^{Bmp4} animals (n = 3 per genotype). E-cad, e-cadherin. Box-and-whisker plot: The *horizontal line* in the middle of each *box* indicates the median; the *top and bottom borders* of the box mark the 75th and 25th percentiles, respectively, the *whiskers* mark minimum and maximum of all the data, and the *circles* indicate outliers. *$P < .05$ by *t* test.

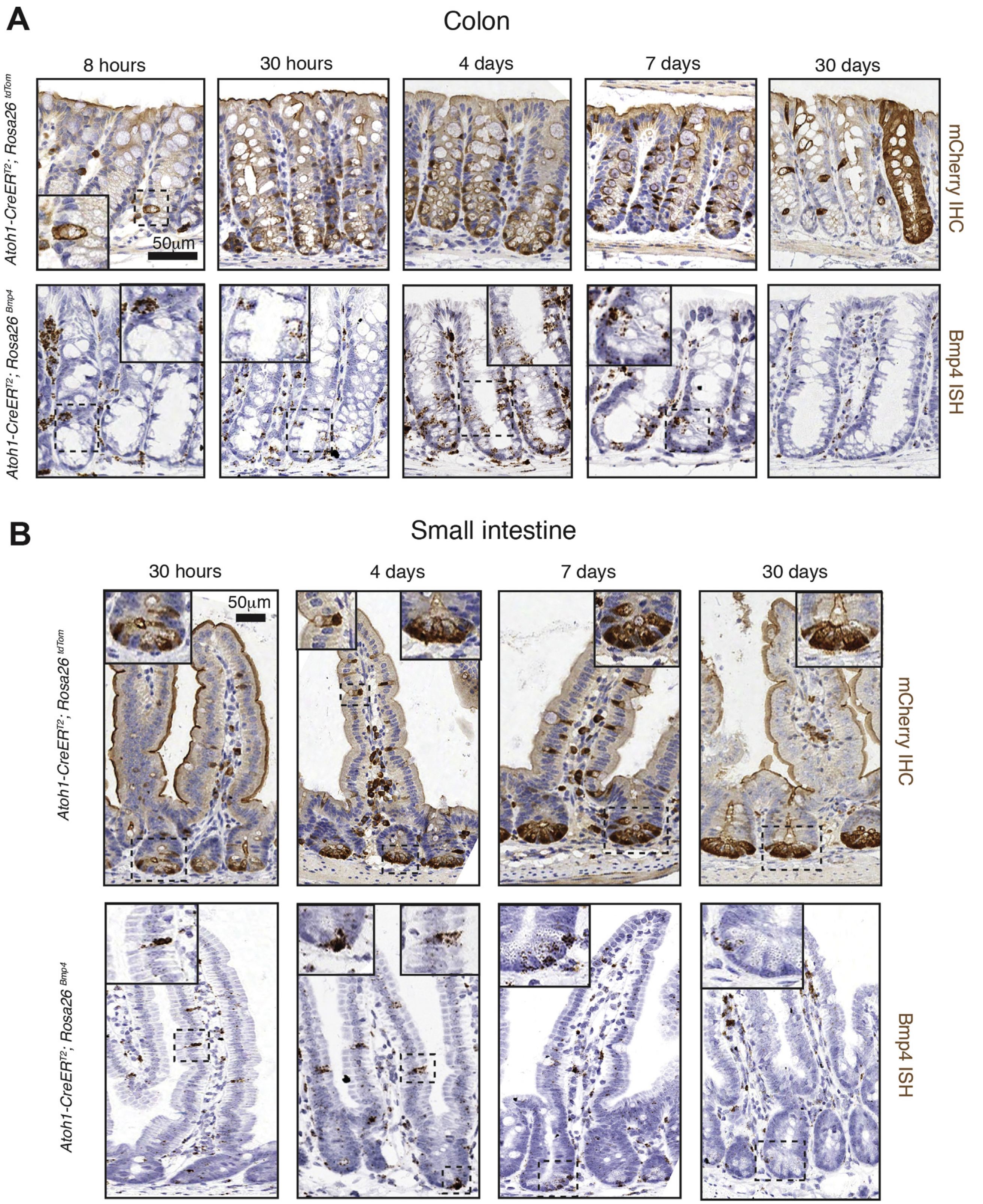

Supplementary Figure 2. Staining for cell counting. Representative images of tdTomato IHC (*brown*) and *Bmp4* ISH (*brown dots*) in secretory epithelial cells of the (*A*) colon and (*B*) small intestine at different times after recombination in *Atoh1-CreER*^{T2};*Rosa26*^{tdTomato} and *Atoh1CreER*^{T2};*Rosa26*^{Bmp4} mice. Scale bar = 50 μm in all panels; *Insets* magnification 400×.

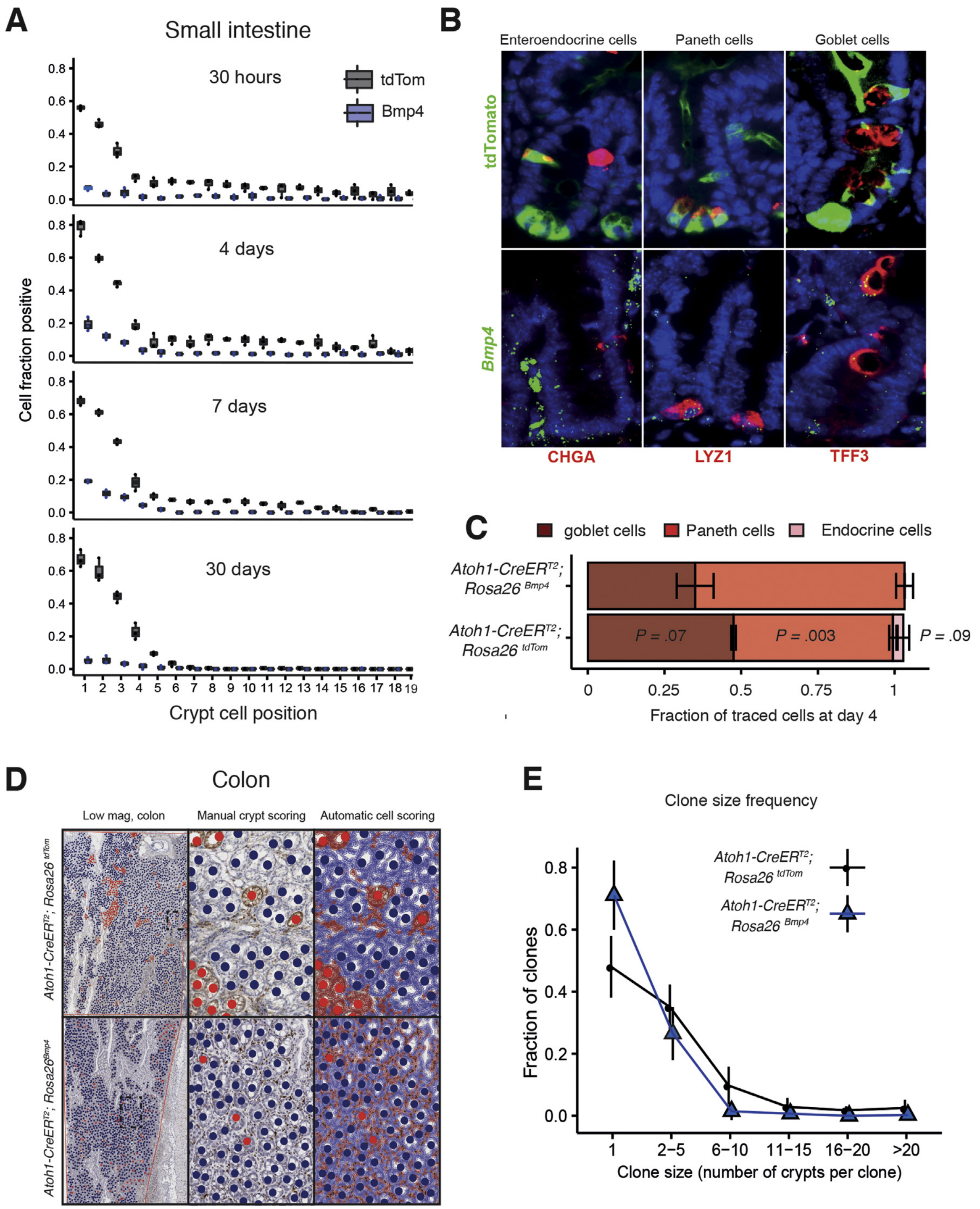

Supplementary Figure 3. Secretory cell model assessment. (*A*) Steady-state small intestinal crypt cell position counts of cells stained with tdTomato IHC (*gray*) or *Bmp4* ISH (*blue*) over time after recombination (n = 3 mice, 7 days: n = 2 mice, 30 crypts counted per mouse). Box-and-whisker plot: The *horizontal line* in the middle of each *box* indicates the median; the *top and bottom borders* of the box mark the 75th and 25th percentiles, respectively, the *whiskers* mark minimum and maximum of all the data. (*B*) Costain of tdTomato IHC (*green*) (*top panels*) or *Bmp4* ISH (*green*) (*bottom panels*) with IHC markers for enteroendocrine cells (chromogranin A [CHGA], *red*), Paneth cells (lysozyme 1 [LYZ1], *red*), and goblet cells (trefoil factor 3 [TFF3], *red*) in the small intestine secretory cell models in steady state, 4 days after recombination. (*C*) Quantification of fraction of traced cells costaining for individual secretory cell markers in different secretory cell models in steady state, 4 days after recombination (n = 3 mice, 9–22 crypts counted per mouse; *t* test, between the same cell types in each genotype, *P* values as stated). (*D*) Representative images of en face sections from secretory cell models taken 30 days after initiation of DSS colitis. Low-power images show variable expansion of clonal patches in different genotypes. High-power images demonstrate manual and automated digital pathology (QuPath) scoring of positive epithelial cell staining, used to exclude noncontributory stromal cell staining. (*E*) Quantification of clone size frequency in different secretory cell models 30 days after DSS initiation shows greater fraction of multicrypt and large clonal patches in *Atoh1CreER*^{T2};*Rosa26*^{tdTom} animals (Rosa26^{Bmp4} n = 5 mice, Rosa26^{tdTom} n = 4 mice). ***P* < .01. The *error bars* indicate the standard deviation.

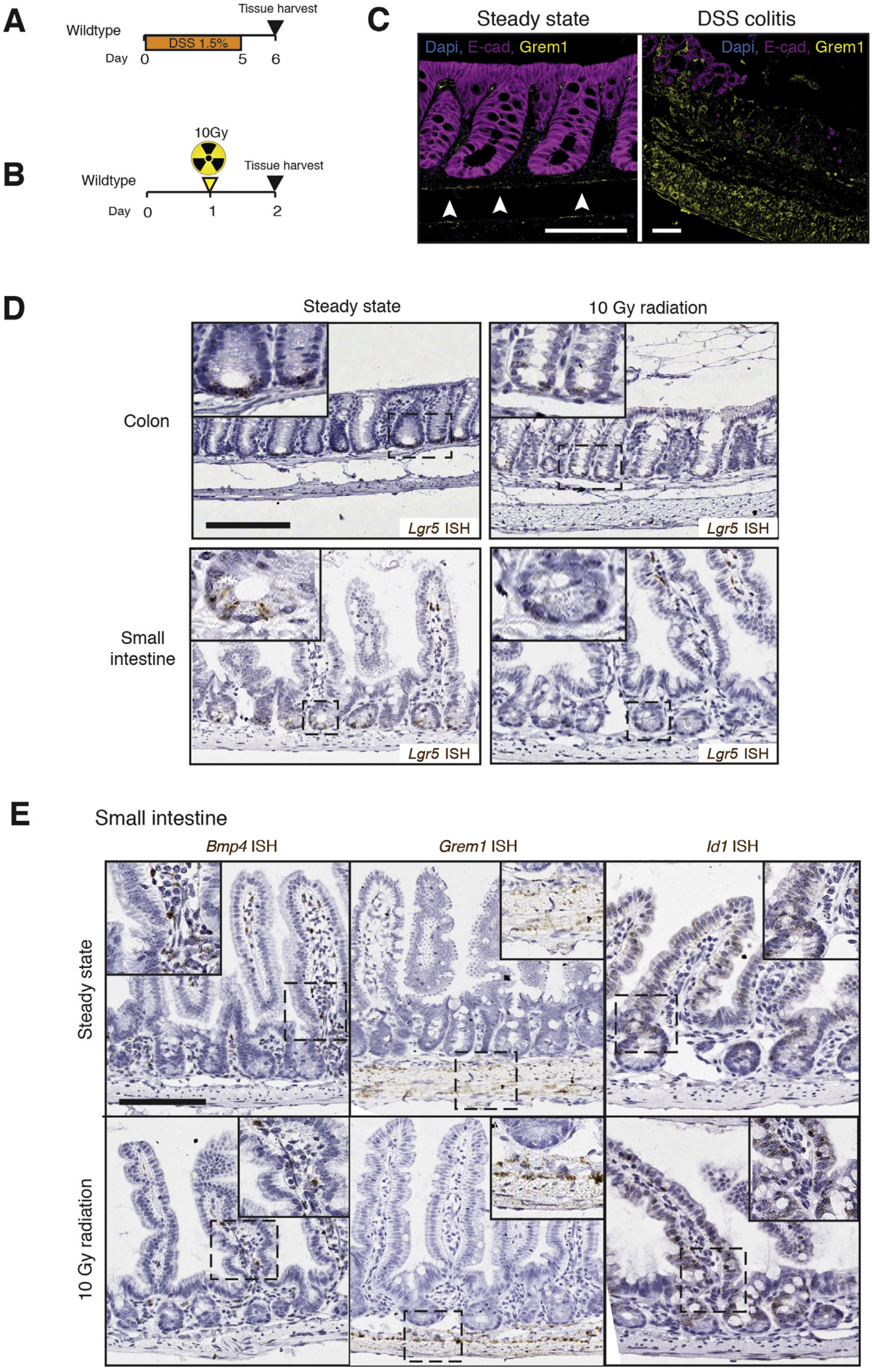

Supplementary Figure 4. Impact of 10 Gy on intestinal regeneration. Schematic shows (*A*) schedule of DSS administration and (*B*) intestinal irradiation and tissue harvesting in animal models of BMP signaling disruption. (*C*) *Grem1* protein (*yellow*) expression in steady-state and DSS colitis mice showing faint staining in the muscularis alone in steady state (*white arrowheads*) and marked increase in expression in ulcerated tissue. (*D*) *Lgr5* ISH shows loss of *Lgr5* expression in crypt base columnar stem cells 24 hours after 10-Gy whole-body irradiation. (*E*) ISH of *Bmp4*, *Grem1*, and *Id1* in mouse small intestine in steady state and after 10-Gy irradiation. Scale bars: 200 μm, magnification applies to all images, except insets.

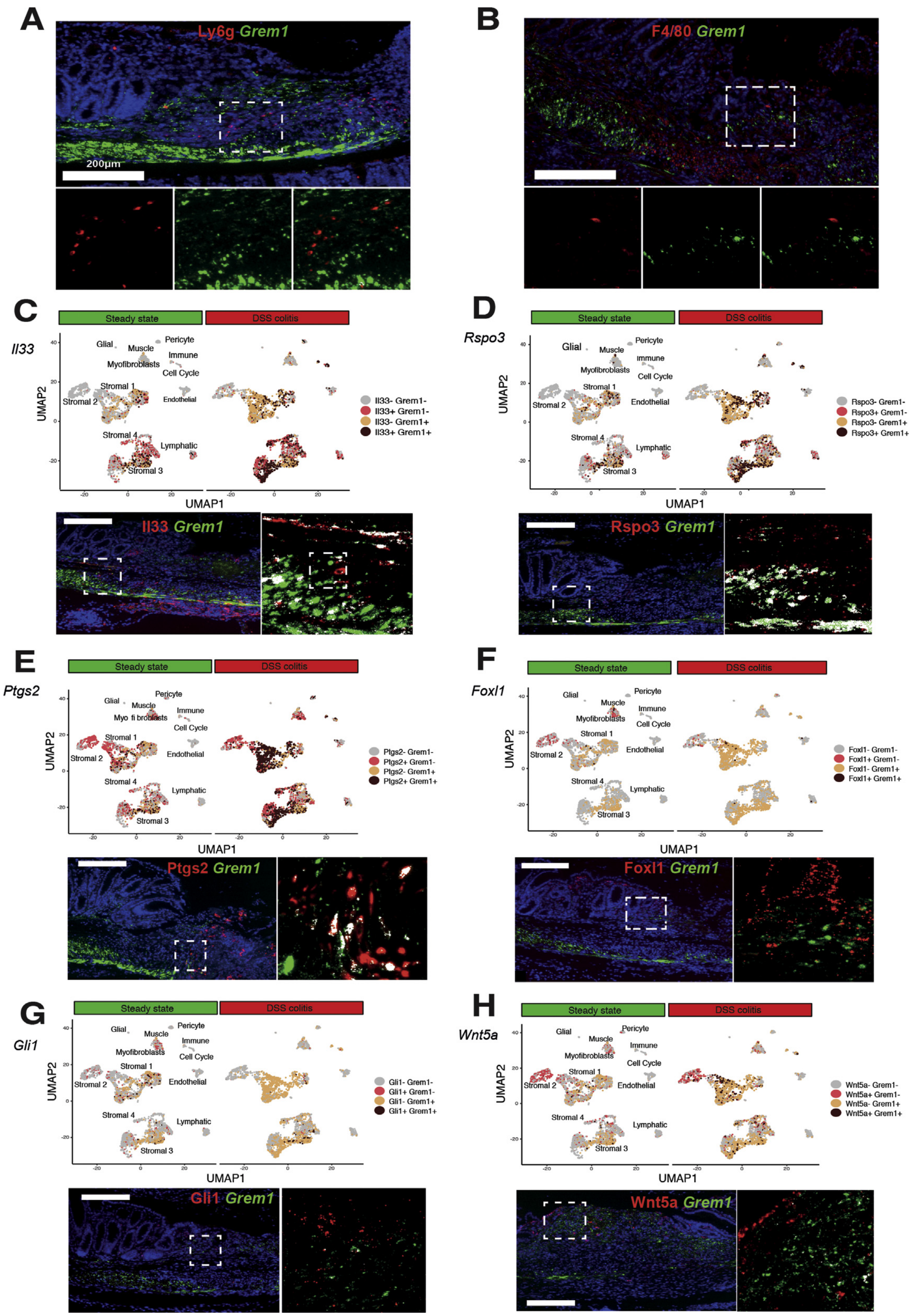

Supplementary Figure 5. Coexpression of *Grem1* with intestinal mucosal immune and stromal cells. There was no overlap of fluorescent expression of *Grem1* mRNA (*green*) with (*A*) neutrophils marked by LY6G (*red*), or (*B*) with macrophages marked by F4/80 (*red*). Single-cell transcription uniform manifold approximation and projection (U-MAP) plots (steady state and DSS colitis) and co-ISH/IHC expression for *Grem1* (*green*) with (*C*) *Il-33* (*red*), (*D*) *R-Spo3* (*red*), and (*E*) Ptgs2 (anti–cyclooxygenase-2 antibody, *red*) showing overlap of expression (*dark brown dots* on U-MAP, *white color* on IHC/ISH) in discrete cells of the muscularis or ulcer bed in intestinal ulcers. Single-cell transcription U-MAP plots (steady state and DSS colitis) and co-ISH showing predominantly distinct cell populations expressing *Grem1* (*green*) and (*F*) *Foxl1* (*red*), (*G*) *Gli1* (*red*), and (*H*) *Wnt5a* in intestinal ulcers. Scale bar = 200 μm in all panels.

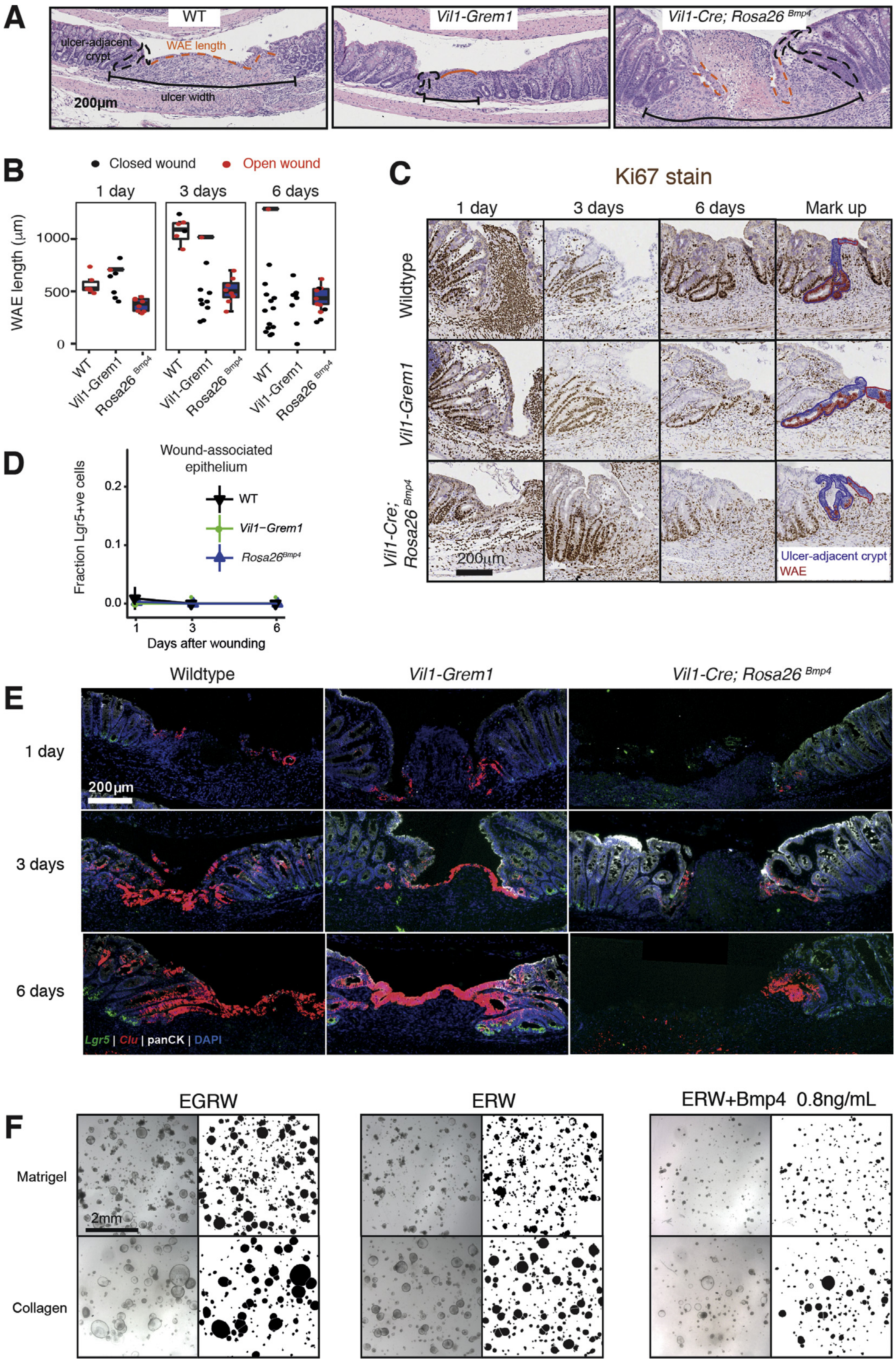
A
WT
ulcer-adjacent crypt
WAE length
ulcer width
200µm
Vil1-Grem1
Vil1-Cre; Rosa26 Bmp4
B
Closed wound
Open wound
1 day
3 days
6 days
WAE length (µm)
1000
500
0
WT
Vil1-Grem1
Rosa26 Bmp4
C
Ki67 stain
1 day
3 days
6 days
Mark up
Wildtype
Vil1-Grem1
Vil1-Cre; Rosa26 Bmp4
200µm
Ulcer-adjacent crypt
WAE
D
Wound-associated epithelium
Fraction Lgr5+ve cells
0.2
0.1
0.0
WT
Vil1-Grem1
Rosa26Bmp4
1
3
6
Days after wounding
E
Wildtype
Vil1-Grem1
Vil1-Cre; Rosa26 Bmp4
1 day
3 days
6 days
200µm
Lgr5 | Clu | panCK | DAPI
F
EGRW
ERW
ERW+Bmp4 0.8ng/mL
Matrigel
Collagen
2mm

Supplementary Table 1. TaqMan Probes Used in Quantitative Reverse Transcription Polymerase Chain Reaction Experiments

Gene	TaqMan probe	Source	Identifier
Bmp2	Mm01340178_m1	Life Technologies	Cat# 4331182
Bmp4	Mm00432087_m1		
Bmp5	Mm00432091_m1		
Bmp7	Mm00432102_ml		
Chrdl1	Mm00473158_m1		
Chrdl2	Mm01136674_m1		
Clu	Mm01197002_m1		
Gli1	Mm00494654_ml		
Grem1	Mm00488615_s1		
Grem2	Mm00501909_m1		
Id1	Mm00775963_g1		
Il6	Mm00446190_m1		
Lgr5	Mm00438890_m1		
Nog	Mm01297833-s1		
Serpine	Mm00435858_ml		
Gapdh		Life Technologies	Cat# 4352661
BMP2	Hs00154192_m1	Life Technologies	Cat# 4331182
BMP4	Hs00370078_m1		
BMP5	Hs00234930_m1		
BMP7	Hs00233476_m1		
CHRDL1	Hs00292767_m1		
CHRDL2	Hs00248808_m1		
GREM1	Hs00171951_m1		
GREM2	Hs03986140_s1		
ID1	Hs03676575_s1		
LGR5	Hs00173664_m1		
NOG	Hs00271352_s1		
GAPDH		Life Technologies	Cat# 4333764F

Supplementary Figure 6. Ulcer quantification and stem cell phenotyping. (*A*) Representative H&E images of 3-day-old biopsy wounding ulcers in different genotype animals, containing examples of ulcer-adjacent crypts (*dashed black lines*), ulcer width (*solid black lines*), and WAE length (*dashed orange lines*). WT, wild-type. (*B*) Dot-and-box plot of WAE length over time in endoscopy biopsy ulcers from WT, *Vil1-Grem1*, and *Vil1-Cre*;*Rosa26*^{Bmp4} animals. Ulcers completely covered with WAE were not included in the boxes, while incompletely covered ulcers are shown as both dots and boxes (n = 6–15 ulcers). The *horizontal line* in the middle of each *box* indicates the median; the *top* and *bottom borders* of the box mark the 75th and 25th percentiles, respectively, and the *whiskers* mark minimum and maximum of all the data. (*C*) Cell staining quantification reveals absence of *Lgr5* expression in wound-associated epithelium over time in all mouse genotypes (n = 3–6 mice per group). (*D*) Representative images of Ki67 stain in different mouse genotypes, with digital pathology cell identification mark up (QuPath) used for quantification of cell proliferation in ulcer-adjacent crypts. (*E*) Representative images of ISH for *Lgr5* (*green*), clusterin (red) with costained IHC for pan-cytokeratin (pan-CK) (*white*) and 4′,6-diamidino-2-phenylindole (DAPI) (*blue*) in the endoscopic biopsy wounds of different genotype animals over time. (*F*) Stereomicroscopy images with organoid area quantification of organoids grown in Matrigel or collagen and treated with media containing variable recombinant proteins (E, epidermal growth factor; G, gremlin 1; R, R-spondin 1; W, Wnt3a). Scale bars = 200 μm in all panels.
